# Bacteriophage: A Useful Tool for Studying Gut Bacteria Function of Housefly Larvae, Musca domestica

**DOI:** 10.1128/spectrum.00599-21

**Published:** 2021-08-11

**Authors:** Xinyu Zhang, Shumin Wang, Ting Li, Qian Zhang, Ruiling Zhang, Zhong Zhang

**Affiliations:** a Collaborative Innovation Center for the Origin and Control of Emerging Infectious Diseases, Shandong First Medical University (Shandong Academy of Medical Sciences), Taian, China; b School of Basic Medical Science, Shandong First Medical University (Shandong Academy of Medical Sciences), Taian, China; University of Minnesota

**Keywords:** housefly larvae, gut microbiota, *Pseudomonas aeruginosa*, bacteriophages, 16S rRNA sequencing, phage gene sequencing

## Abstract

Beneficial symbiotic bacteria have positive effects on some insects’ (such as mosquitoes, cockroaches, and flies) biological activities. However, the effects of a lack of one specific symbiotic bacterium on the life activities of some insects and their natural gut microbiota composition remain unclear. Bacteriophages are viruses that specifically target and kill bacteria and have the potential to shape gut bacterial communities. In previous work, Pseudomonas aeruginosa that naturally colonized the intestines of housefly larvae was shown to be essential to protect housefly larvae from entomopathogenic fungal infections, leading us to test whether a deficiency in Pseudomonas aeruginosa strains in housefly larvae that was specifically caused using bacteriophages could remold the composition of the intestinal bacteria and affect the development of housefly larvae. Our research revealed that the phage, with a titer of 10^8^ PFU/ml, can remove 90% of Pseudomonas aeruginosa in the gut. A single feeding of low-dose phage had no effect on the health of housefly larvae. However, the health of housefly larvae was affected by treatment with phage every 24 h. Additionally, treating housefly larvae with bacteriophages every 24 h led to bacterial composition changes in the gut. Collectively, the results revealed that deficiency in one symbiotic gut bacteria mediated by precise targeting using bacteriophages indirectly influences the intestinal microbial composition of housefly larvae and has negative effects on the development of the host insect. Our results indicated the important role of symbiotic gut bacteria in shaping the normal gut microbiota composition in insects.

**IMPORTANCE** The well-balanced gut microbiota ensures appropriate development of the host insect, such as mosquitoes, cockroaches, and flies. Various intestinal symbiotic bacteria have different influences on the host gut community structure and thus exert different effects on host health. Therefore, it is of great importance to understand the contributions of one specific bacterial symbiont to the gut microbiota community structure and insect health. Bacteriophages that target certain bacteria are effective tools that can be used to analyze gut bacterial symbionts. However, experimental evidence for phage efficacy in regulating insect intestinal bacteria has been little reported. In this study, we used phages as precision tools to regulate a bacterial community and analyzed the influence on host health after certain bacteria were inhibited by bacteriophage. The ability of phages to target intestinal-specific bacteria in housefly larvae and reduce the levels of target bacteria makes them an effective tool for studying the function of gut bacteria.

## INTRODUCTION

Insect guts provide proper environments for microbial colonization, and bacteria in the gut potentially provide several benefits to their hosts (1). The intestinal microbiota, which contains bacteria, archaea, viruses, and fungi (2, 3), directly and indirectly affects the health and fitness of many insects (4). Although in some species, such as *Lepidoptera* (caterpillars), the gut microbiome is not essential and suppressing gut bacteria has no detectable effect on caterpillar growth or survival (5), the gut microbiota participates in the life activities of most insects (6) and plays important roles in many physiological functions, such as nutrition, metabolism, and immunity (7). Disruption of the normal microbiota would lead to increased mortality in most insects. Studies have shown that Pseudomonas species in insects participate in detoxification (8) and digestion (9, 10) activities. The intestinal microbiota of insects invaded by Pseudomonas undergoes significant changes, resulting in insect disease and death (11). However, these studies mainly deal with the influence of specific bacteria or their excess on the development of host insects. There are few reports and analysis of changes in the gut microbiota and host health caused by the lack of a certain bacterium as a result of bacteriophage infection. Studies have reported research using Gram-negative bacteria-targeting antibiotics to remove bacteria in the intestines of housefly larvae to analyze the role of Pseudomonas aeruginosa during Beauveria bassiana infection (12); however, the use of antibiotics decreased the abundances of other Gram-negative bacteria in the intestine. Therefore, the benefits of Pseudomonas aeruginosa alone cannot be effectively demonstrated. Bacteriophages have a powerful targeting ability that allows them to remove specific bacteria.

Bacteriophages are the top predators of the bacterial world. As specific bacterial predators, they increase microbial diversity through Red Queen/kill-the-winner dynamics (13, 14). In particular, researchers have reported the role of bacteriophages in shaping bacterial communities and highlighted their immense diversity and potential role in animal (15) and plant (16) bacteriophage therapy research. However, the interactions of specific bacteriophages with their hosts and their influence on host gut communities are still poorly understood. In most ecosystems, bacteriophages are the most common and diverse biological entities (17). Studies on the human gut ecosystem show that free phages work as an intestinal barrier, maintaining the balance of intestinal flora by controlling invasive bacterial populations (18). Phages have been utilized as curative elements in bacterial infection treatment in various fields, including aquaculture (19), agriculture (20), and human clinical trials (21). The pathogen-specific viruses used in phage therapy provide a more effective method for manipulating the microbiota and protecting animals and plants from various diseases (22). Compared with antibiotics and bacterial inoculants, phages are highly host specific and propagate rapidly in the presence of host bacteria with little influence on other bacteria. Therefore, phages could be used as precision tools to target and kill pathogens, leaving the nontargeted microbiota unaffected (20, 23).

Bacteriophages regulate bacterial density and affect bacterial diversity by restricting the expression of bacterial virulence genes (24) and disrupting bacterial bacteriophage defense systems (25, 26). Moreover, phage-mediated reduction in pathogen density could indirectly affect the diversity and function of other intestinal microbes (27). Bacteriophages reduce the density of a certain bacterium and increase the habitable space and available nutrients of other microorganisms. In addition, phages weaken the ability of the targeted bacterium to compete with other microorganisms for living resources, thereby further reducing the density of this bacterium (28–30). Eventually, these changes lead to changes in microbial components and diversity. Both the regulation of bacterial density and coevolution by bacteriophages may have an impact on the health of insects because changes in the composition of the microbial group are usually associated with the incidence of disease (31, 32). Phages could be used to manipulate insect gut bacterial communities to promote the health of bacterial infection insect models (33). In the insect (Galleria mellonella) model of Clostridium difficile colonization, Nale et al. (34) observed that preventive treatment with phage resulted in 100% survival, while application of phage 2 h after bacterial challenge resulted in 30% survival. Mikonranta et al. used cabbage looper (Trichoplusia ni) larvae (35), an insect model, to study the interaction between bacteriophages and antibiotics and finally clarified that bacteriophage can assist in targeting antibiotic-resistant bacteria. The work of Nale et al. and some research by others further highlight the therapeutic potential of bacteriophage in insects (35–37). However, established experimental models often focus on single bacteria-phage pairs in the natural environment, whereas interactions between bacteriophages and the total intestinal environment could be more complex, possibly being affected by the host immune system (38, 39), spatial structures within the tissues, nutrient availability (39), and the native microbial microbiota. Therefore, experimental insect gut microbiota models need to be established to study the interactions between phages, their host bacteria, and the total intestinal colonizing microorganisms. These studies expand our understanding of the interaction between insect health and beneficial symbiotic bacteria in the intestinal environment.

Here, we present an insect gut model to study the effect of inhibiting certain bacteria from intestines on the growth and development of the host insect. Previous study has reported that low concentration P. aeruginosa Y12 could protect housefly larvae from entomopathogenic fungal infection through the production of antifungal metabolites (12). We sought to isolate phages that target Pseudomonas aeruginosa from the natural environment. In this study, the efficiency of bacteriophages in the inhibition of specific bacteria and the influence on growth and development of housefly larvae after certain bacterial deficiencies were achieved were analyzed through bacteriophage feeding experiments. To further analyze whether the invasion of bacteriophages changed the composition of host intestinal microbes and whether the phage-mediated reduction in Pseudomonas aeruginosa causes intestinal bacteria imbalance, the community structure and bacterial diversity of intestinal bacteria were investigated through 16S rRNA gene analysis using a high-throughput sequencing method. The results of this study provide valuable insights into how changes in the abundance (using phages to reduce the number of bacteria) of a single bacterium play a role in the change in the interactions of the intestinal flora and health of insects.

## RESULTS

### Isolation of bacteriophages from lake water targeted against Pseudomonas aeruginosa of housefly larval intestine.

**FIG 1:**
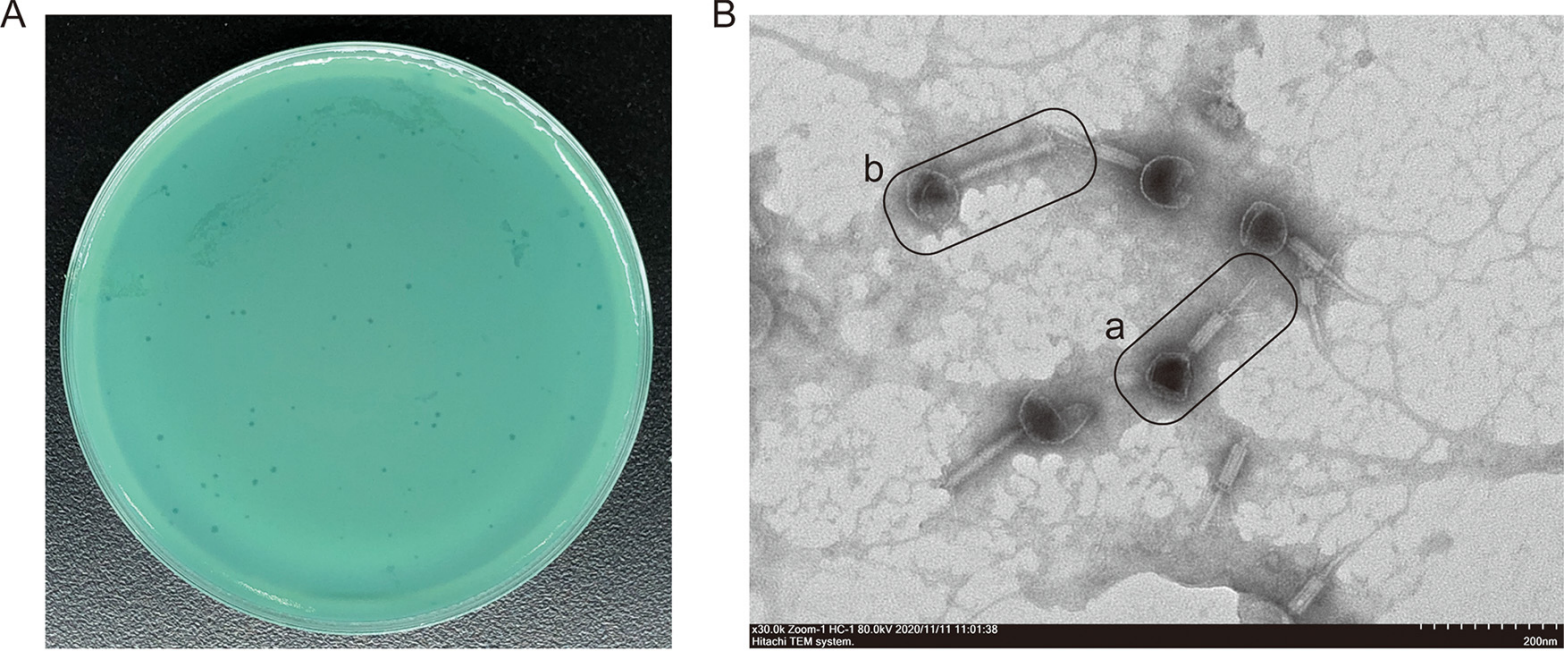
The morphology of Pseudomonas aeruginosa phage Y12Pw and plaque morphology. (A) Morphology of bacteriophage plaques in nutrient agar medium. The plaques of Y12Pw are medium in size and transparent. (B) Electron micrograph of negatively stained, purified bacteriophages used in this study. Form a shows Y12Pw with uncontracted tails and an empty head. Form b shows a contracted tail with straight tail fibers.

**FIG 2:**
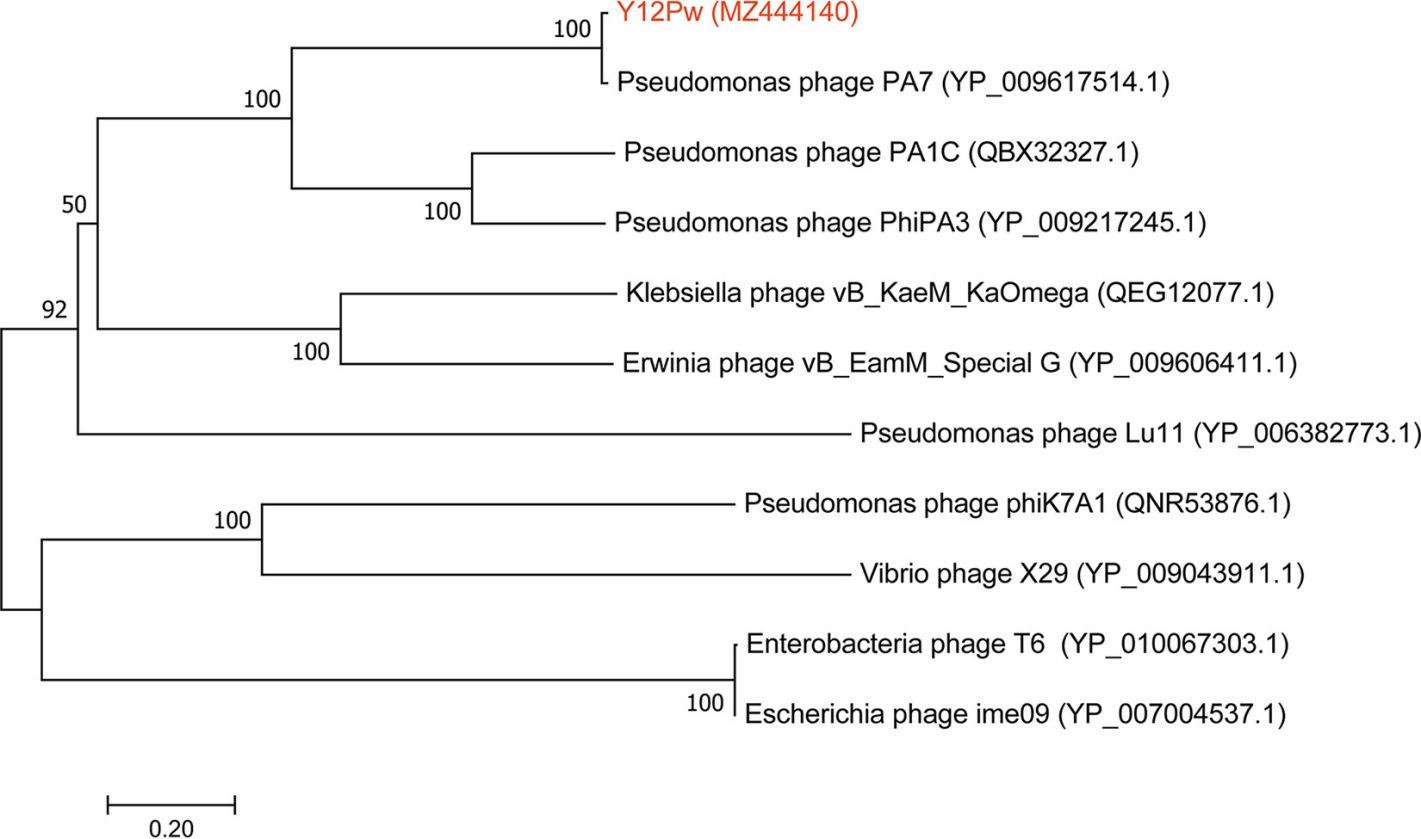
A neighbor-joining phylogenetic tree showing the relatedness of phage Y12Pw with similar phages publicly available in NCBI. A neighbor-joining phylogenetic tree based on tail fiber protein similarity between the phage used in this experiment (red color) and other similar phages that are publicly available at NCBI (black color). The scale bar represents 0.2 amino acid substitutions per site, and values next to the nodes show bootstrap values based on 500 samples.

**FIG 3:**
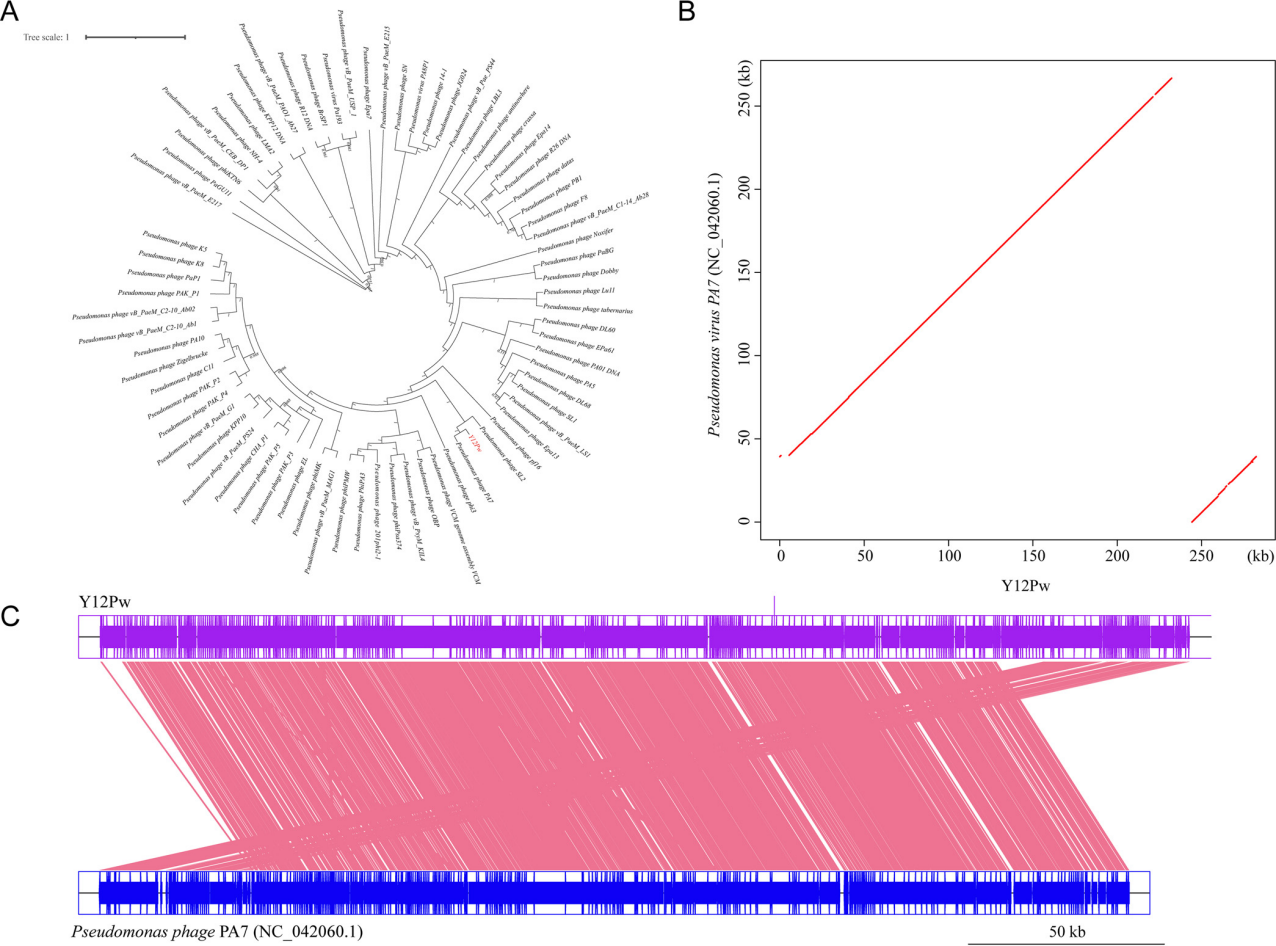
Collinearity analysis of the complete genome between Y12Pw and Pseudomonas phage PA7. (A) A maximum likelihood phylogenetic tree based on genome sequence similarity between the phages used in this experiment (red color) and similar phages (black color) that are publicly available at NCBI. Y12Pw (GenBank accession number MZ444140) belongs to the order *Caudovirales* and is highly similar (>99.9%) to Pseudomonas phage PA7 in the family *Myoviridae* (GenBank accession number NC_042060.1). (B) Two-dimensional comparison between two genomes. The vertical and horizontal axes represent the Pseudomonas phage PA7 genome and reference Y12Pw genome, respectively. (C) Two genomes on parallel collinearity. The upper and lower shafts represent the sequenced (Y12Pw) and reference genomes (Pseudomonas phage PA7), respectively.

We isolated the bacteriophage Y12Pw from the river water of Tai’an City (Table S1 in the supplemental material) through the double-layer agar method. The bacteriophage Y12Pw has a good lysing effect on Pseudomonas aeruginosa Y12 (Fig. 1A). When the phage lyses the bacteria on the plate medium, a plaque is formed, which is counted as a plaque. The PFU refers to the number of infectious phages contained in each milliliter of sample. The bacteriophage Y12Pw presented a latency period of 20 min, with a burst size of approximately 210 particles/infected cell. (Fig. S1A). The bacteriophage demonstrated a narrow intraspecies host range and could not infect any of the other nine strains isolated from housefly larval intestine, including Enterobacter hormaechei, Klebsiella pneumoniae, Acinetobacter bereziniae, Providencia stuartii, Enterobacter cloacae, Lactococcus lactis, Lysinibacillus fusiformis, Providencia vermicola, and Bacillus safensis (Fig. S1B). As observed by transmission electron microscopy (Fig. 1B), form a has an uncontracted tail and an empty head, and form b shows a contracted tail with straight-tailed fibers. The head of the phage has an icosahedral structure with a retractable tail and belongs to the *Myoviridae* family. After genome sequencing analysis, the genome of Y12Pw has 282,378 bp with an average GC content of 36.82% (Fig. S2). A neighbor-joining phylogenetic tree (Fig. 2) showed that Y12Pw (GenBank accession number MZ444140) belonged to the order *Caudovirales* and family *Myoviridae* and was highly similar with Pseudomonas phage PA7 (>99% similarity) (GenBank accession number NC_042060.1). Then, we performed a whole-genome comparison analysis of Y12Pw and PA7. A maximum likelihood phylogenetic tree showed that Y12Pw was similar to PA7 (>99.9% similarity) (Fig. 3A). Y12Pw genomes and Pseudomonas phage PA7 genomes were closely related to each other (Fig. 3B). Their genes are highly homologous, with clear collinearity (Fig. 3C). Through VIRFAM analysis, we found that the bacteriophage K139 (belonging to family *Myoviridae*) (40) was similar to the head-neck-tail module of Y12Pw (Fig. S3).

### The effects of phages targeting Pseudomonas aeruginosa in the intestine on the growth and development of housefly larvae.

**FIG 4:**
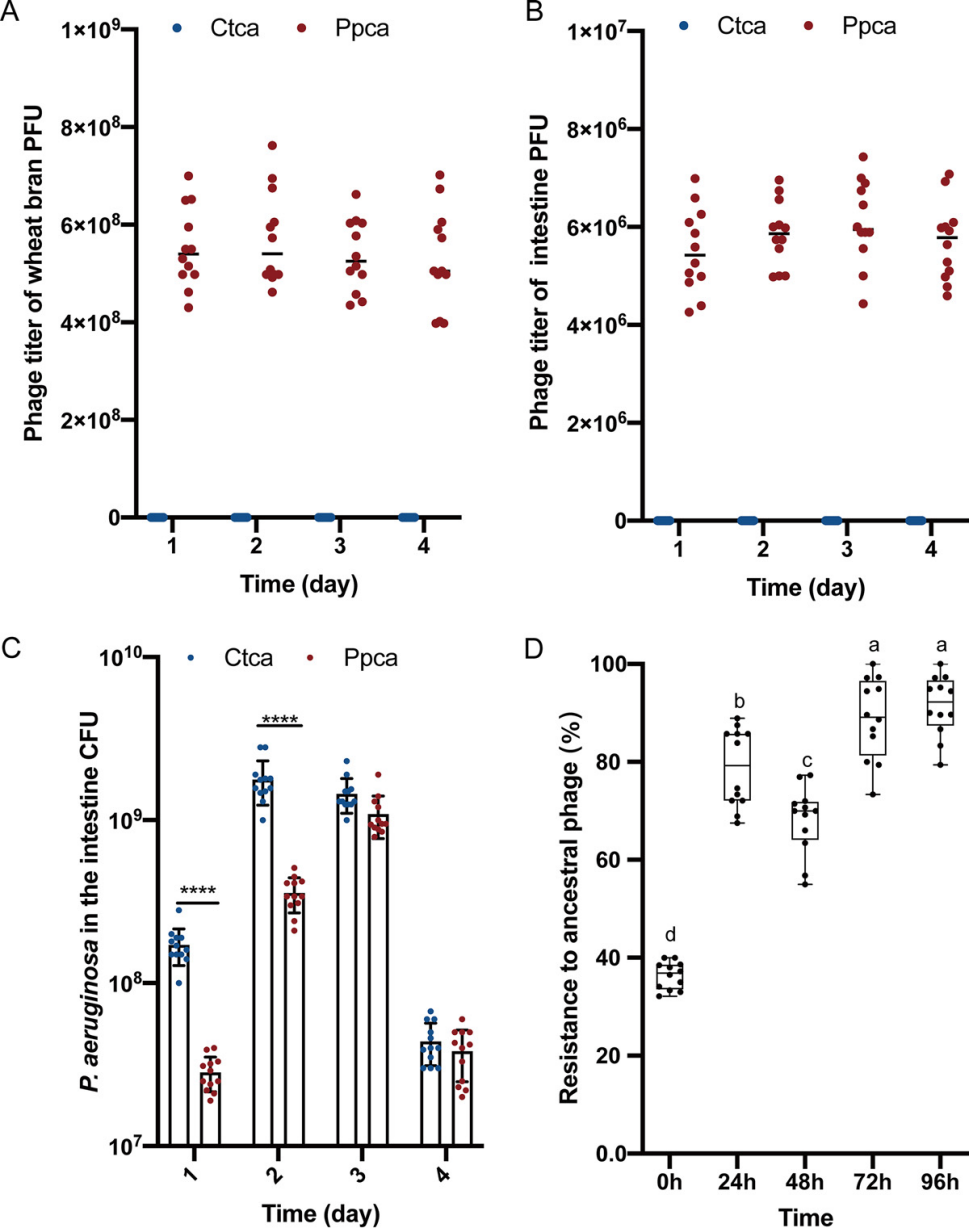
PhageY12Pw that survive well both *in vivo* and *in vitro* can reduce their host bacteria *in vivo* (P. aeruginosa that has evolved resistance to ancestor phages). Phage titer significantly changed over time in wheat bran (A) (upon which housefly larvae breed) and in housefly larval intestines (B) with different treatments. Ctca and Ppca represent housefly larvae samples treated with sterile water and sterile water containing 10^8^ PFU/ml bacteriophage, which was replaced every 24 h, respectively. (C) Pseudomonas aeruginosa in housefly larval gut samples of the Ppca group and Ctca group was determined by plating on selective medium. Ctca and Ppca represent housefly larvae samples treated with sterile water and sterile water containing 10^8^ PFU/ml bacteriophage, which was replaced every 24 h, respectively. Data are shown as the mean ± standard error of the mean (SEM). Repeated measures ANOVA was followed by Sidak correction for multiple comparisons; *, *P < *0.05; **, *P < *0.01; ***, *P < *0.001. (D) The mean phage resistance to ancestral and coevolved phages after 24 h of growth; *n* = 12 for the control (0 h) (original strains that have not been infected with phage), and *n* = 12 for single phage treatment every 24 h (bacteria strains that infect ancestral phages and coevolving phages). Multiple comparisons were conducted using a Tukey test and a false-discovery rate (FDR)-adjusted *P *value of <0.05.

**FIG 5:**
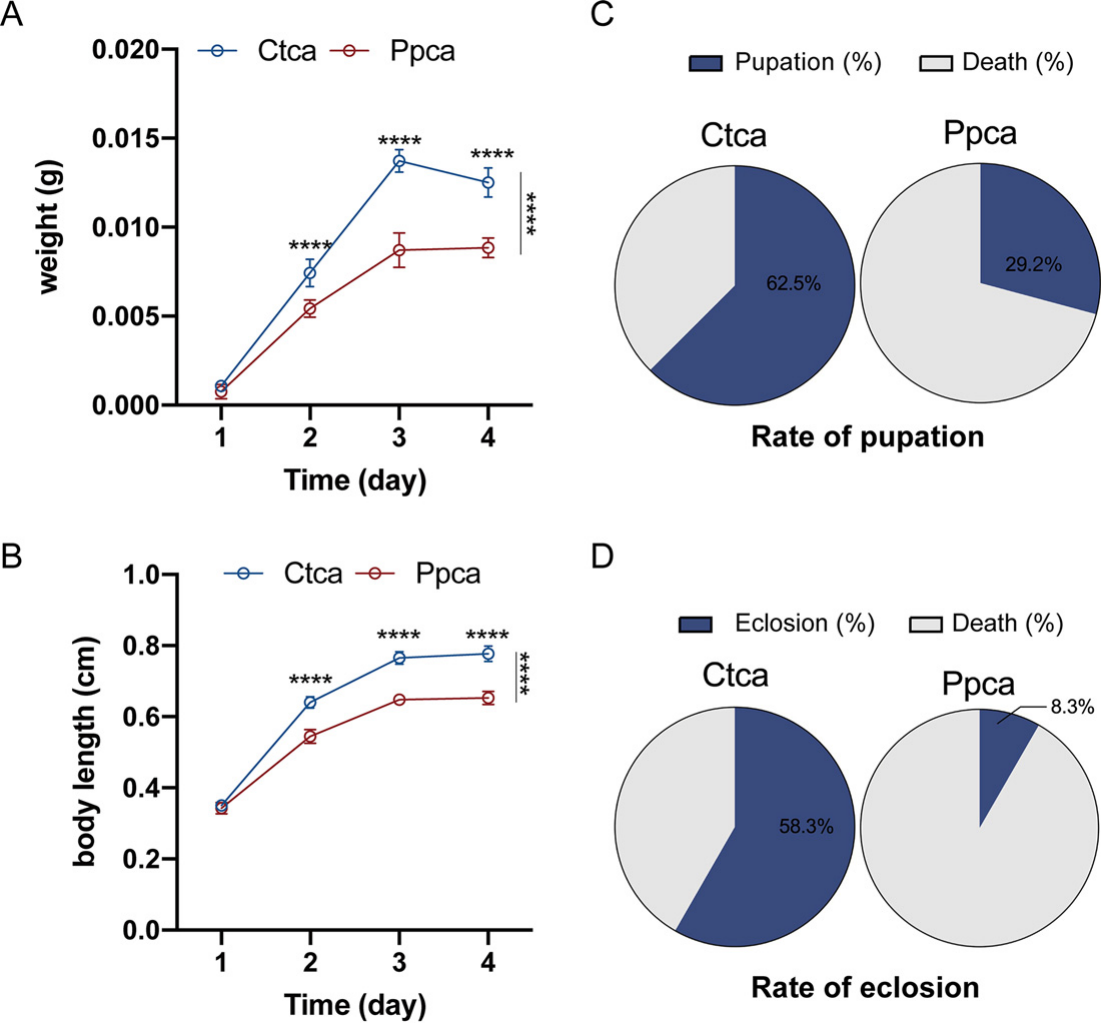
Changes in the developmental duration of housefly larvae treated with sterile water and sterile water containing 10^8^ PFU/ml bacteriophage. (A) Body weight (ANOVA, main effect of time, *F*_3,66_ = 1,196 and *P < *0.0001; main effect of diet, *F*_1,22_ = 567.0 and *P < *0.0001; *n* = 12; time × diet interaction, *F*_3,66_ = 54.54 and *P < *0.0001) and (B) body length (ANOVA, main effect of time, *F*_3,66_ = 2,803 and *P < *0.0001; main effect of diet, *F*_1,22_ = 395.8 and *P < *0.0001; *n* = 12; time × diet interaction, *F*_3,66_ = 68.24 and *P < *0.0001) significantly changed over time in housefly larvae with different treatments. The pupation rates (C) and eclosion rates (D) of housefly larvae were significantly different in the treatment groups. Ctca and Ppca represent housefly larvae samples treated with a sterile water diet and sterile water containing 10^8^ PFU/ml bacteriophage that was replaced every 24 h, respectively. Data are shown as the mean ± SEM. Repeated measures ANOVA was followed by a Sidak correction for multiple comparisons; *, *P < *0.05; **, *P < *0.01; ***, *P < *0.001.

To analyze whether bacteriophage Y12Pw can exist stably and target its host bacteria in intestine, we propose a novel housefly larval intestine model. Based on this purpose, we concentrated the purified phage and adjusted the phage titers to 10^8^ phage particles per ml, and phage stocks were stored at 4°C. We chose newly hatched housefly larvae as experimental animals and subsequently treated them with phage Y12Pw every 24 h. We then used a plate plaque experiment to detect the titers of bacteriophage Y12Pw in the intestines and in wheat bran in the intestines of housefly larvae in a phage treatment (Ppca) group and a control (Ctca) group. Our results revealed that bacteriophages can maintain good titers in the complex intestinal environment of housefly larvae and in the wheat bran upon which they breed (Fig. 4A and B). To verify that phage Y12Pw was able to reduce the levels of Pseudomonas aeruginosa *in vivo*, Pseudomonas aeruginosa in the gut in the Ppca group and Ctca group was determined by plating on selective medium. We continuously monitored the changes in Pseudomonas aeruginosa in larval intestine for 4 days (Fig. 4C). Continuous addition of bacteriophages to housefly-breeding wheat bran persistently suppressed P. aeruginosa growth in the intestine for 2 days (Fig. 4C). However, phage target P. aeruginosa that exhibit increased resistance to ancestral phages (*F*_4,55_ = 3.569, *P < *0.05), especially on the third and fourth day. (Fig. 4D). The results showed that bacteriophages can effectively remove 90% of Pseudomonas aeruginosa in the intestines of housefly larvae, leading to the lack of these bacteria in the intestines. Moreover, the growth and development of housefly larvae treated with phage were negatively affected. Specifically, on the second day, housefly larvae began to grow slowly, and their body length and body weight were lower than those of the control group (Fig. 5A and B). Moreover, the pupation rate (29.2%) and eclosion rate (8.3%) of housefly larvae in the Ppca group were also significantly affected and were lower than those in the control group (rate of pupation, 62.5%; rate of eclosion, 58.3%) (Fig. 5C and D). Phages are viruses that infect only bacteria. Therefore, we tested the safety of bacteriophages against housefly larvae by feeding housefly larvae less than 10^8^ PFU/ml phage only once. Our experiment shows that the housefly larvae of the Ppsa group fed a single feeding of the phage group grew well (Fig. S4). Together, these results suggest that bacteriophages can perform their functions in the intestines of housefly larvae and that the undesirable results may be caused by the decrease in Pseudomonas aeruginosa and the indirect feedback changes of the intestinal bacteria.

### The effect of phage-mediated lack of certain bacteria on the housefly larva intestine microbiota.

**FIG 6:**
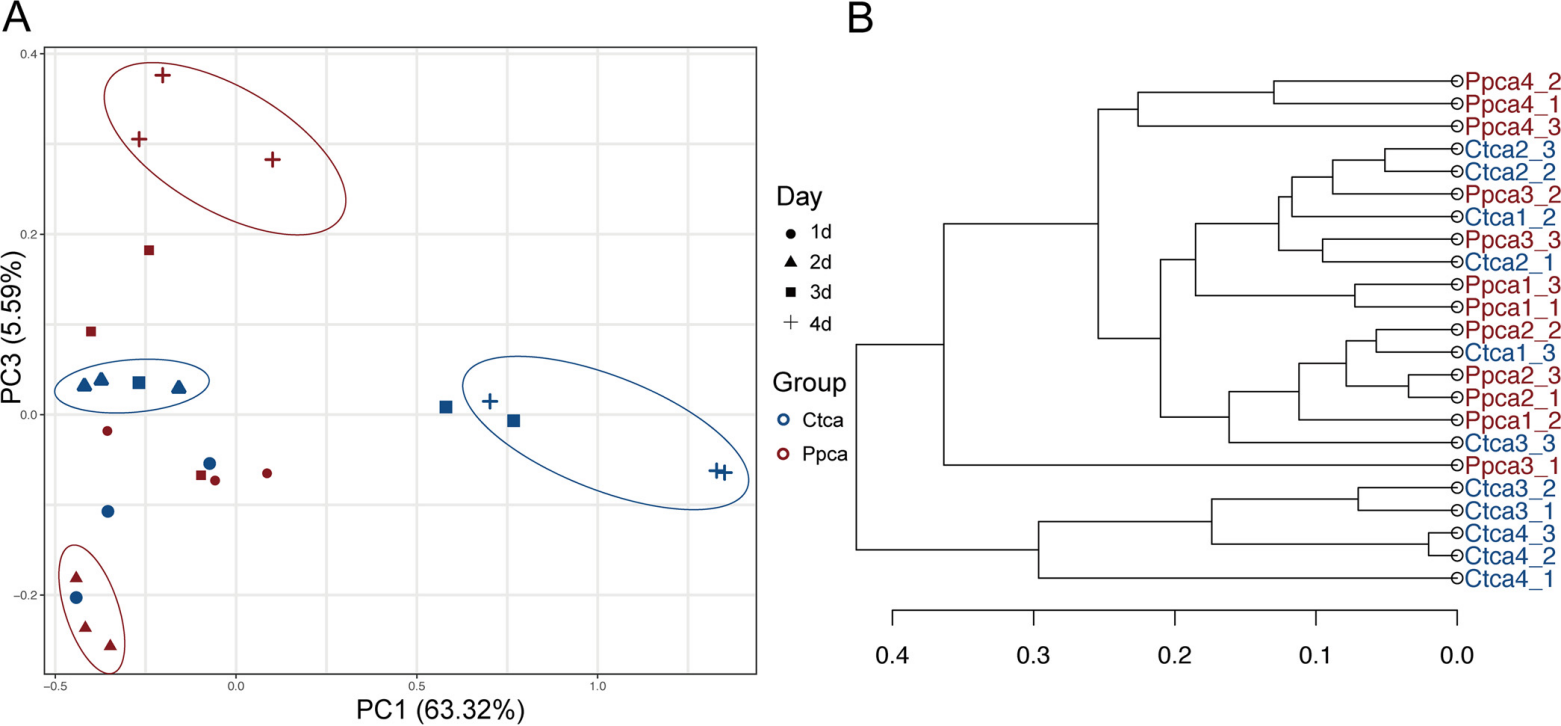
Principal coordinate analysis (PCoA) distinguishing the OTU profiles for all the gut bacteria of housefly larvae samples. (A) PCoA of bacterial community structures of the two groups. Each symbol represents one sample of intestinal bacteria. (B) UPGMA tree analysis of samples in evolution.

We analyzed the intestinal specimens of housefly larvae in the Ppca group and the Ctca group from day 1 to day 4, naming 12 samples Ctca1, 2, 3, and 4 and Ppca1, 2, 3, and 4. To examine the effects of phage-mediated reduction of Pseudomonas aeruginosa on bacterial community composition, housefly larval intestinal bacterial 16S rRNA genes were sequenced (BioProject ID PRJNA746668), yielding a total of 1,473,263 high-quality bacterial sequences with sequence numbers ranging from 52,065 to 74,963 per sample, and these sequences were normalized and clustered into 2,594 operational taxonomic units (OTUs) at a 97% similarity level among all the samples (Table S2). Group Ctca and Ppca shared 164 bacterial genera. The Chao1, Simpson, and Shannon indices suggested that the presence of bacteriophages did not change the bacterial diversity and richness in the Ppca group (Fig. S5A). There were 283 common genera of bacteria in the two groups of housefly intestines (Fig. S5B and C). Notably, Pseudomonas aeruginosa deficiency had a great effect on the composition of the gut microbiome (Fig. 6). The first dimension of the principal-coordinate analysis (PCoA) explained 63.32% of the total variation in the bacterial community. The third dimension of the PCoA explained an additional 5.59% of total variation in the bacterial community. A PCoA showed that the composition of the intestinal bacteria began to show differences after 2 days of treatment, and these differences became more significant at the end of the experiment (Fig. 6A). An unweighted pair-group method with arithmetic mean (UPGMA) tree revealed that Ppca4 is not clustered with Ctca4, which was consistent with the PCoA results (Fig. 6B). Therefore, the disturbance of the intestinal bacteria caused by Pseudomonas aeruginosa bacteriophage treatment became more obvious after 2 days.

**FIG 7:**
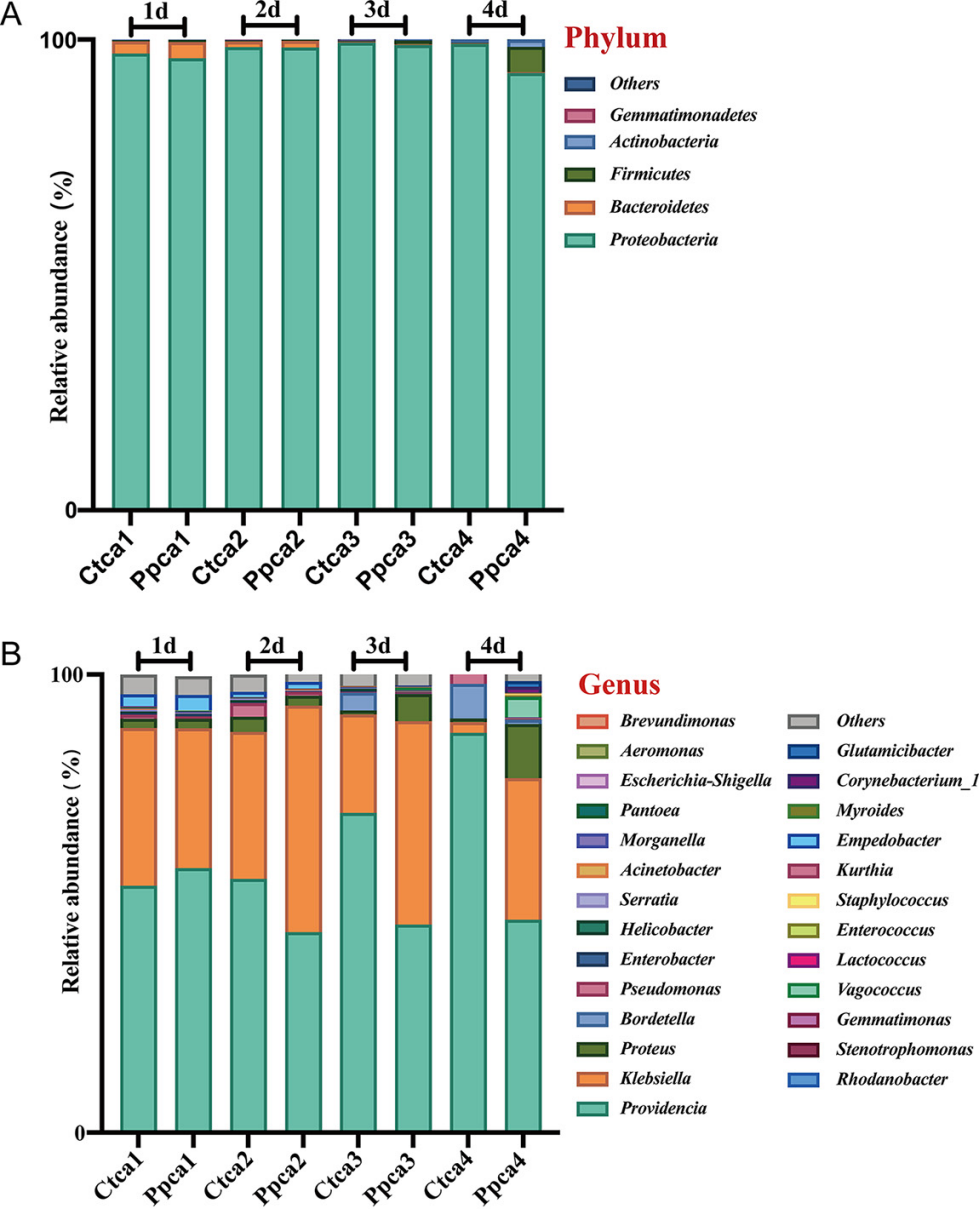
Relative abundances of the top 5 phyla and the top 26 genera of intestinal bacteria in phage-infected housefly larvae and the control group. (A) Relative abundances of the top 5 phyla in housefly larval samples. (B) Relative abundances and distributions of the top 26 genera in housefly larval samples. Bacterial phyla and genera with abundances of over 1% in at least one sample are defined as major phyla and genera. Phyla and genera with percentages lower than 1% in all samples were defined as minor phyla and genera.

Here, we selected the top 5 phyla and the top 26 genera with relatively high abundances for analysis. The results showed that the housefly larvae treated by different strategies had different bacterial community structures at genus levels, and there is only a slight difference between the 3rd day and the 4th day at the phylum level (Fig. 7A and B). *Proteobacteria* had the highest percentage across all the intestinal bacteria of housefly larvae samples, accounting for 98.54% and 96.51% of the total sequences retrieved on average for the Ctca and Ppca groups, respectively (Fig. 7A). In addition, within 3 to 4 days after phage treatment, the proportion of *Firmicutes* (Ppca3d: *t* = 6.623, *P < *0.05; Ppca4d: *t* = 6.859, *P* < 0.05) and *Actinobacteria* (Ppca3d: *t* = 3.858, *P < *0.05; Ppca4d: *t* = 1.691, *P* > 0.05) increased (Fig. 7A). Among the 26 most abundant genera in the housefly larval intestine, Proteus (Pp3d: *t* = 4.350, *P* < 0.05; Pp4d: *t* = 5.357, *P < *0.05), Klebsiella (Ppca2d: *t* = 5.390, *P < *0.05; Ppca4d: *t* = 4.097, *P < *0.05), and *Vagococcus* (Ppca3d: *t* = 7.355, *P < *0.05; Ppca4d: *t* = 6.600, *P < *0.05) in the Ppca group increased greatly in abundance, but *Bordetella* (Ppca2d: *t* = 5.582, *P < *0.05; Ppca3d: *t* = 5.493, *P < *0.05) showed a significantly decreasing trend (Fig. 7B). Therefore, although the bacteriophage specifically targets Pseudomonas aeruginosa in the intestinal tract, it interacts with other intestinal bacteria indirectly, so the composition of the intestinal bacterial community of housefly larvae is greatly disturbed.

**FIG 8:**
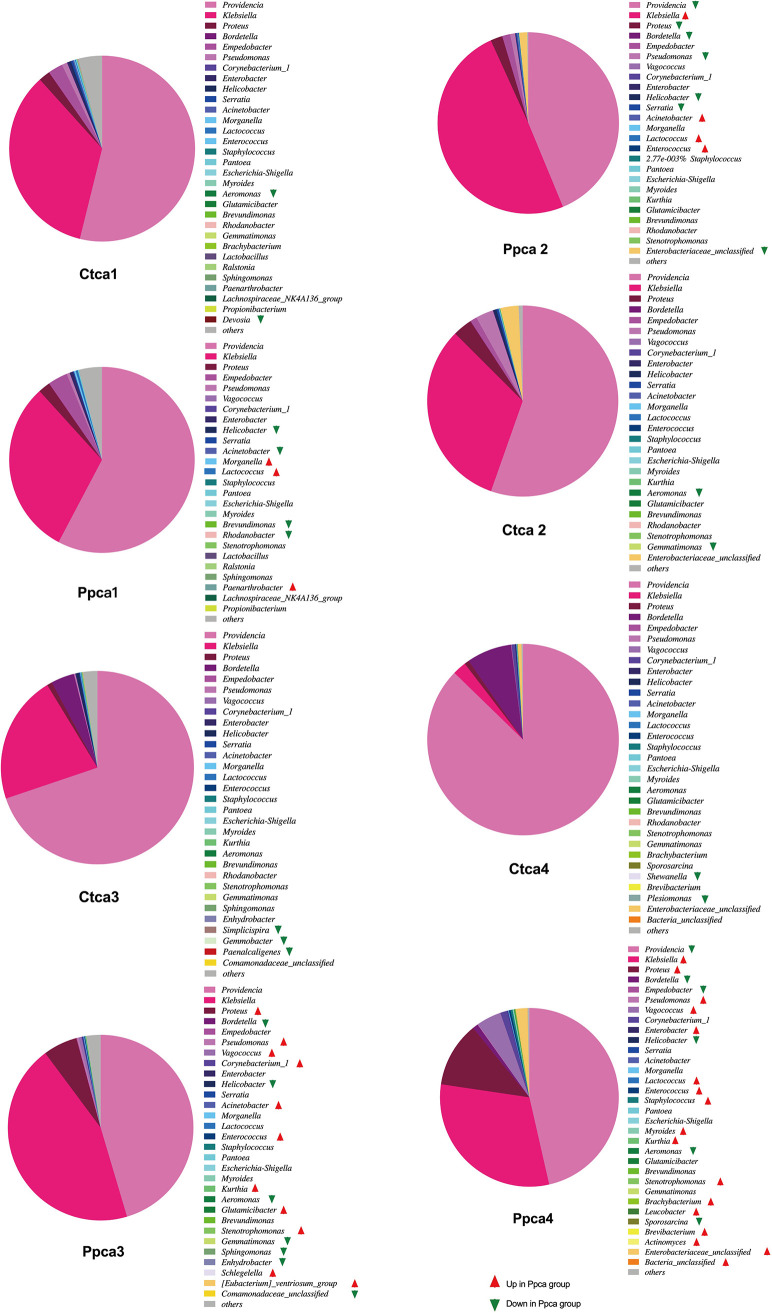
Bacterial genera with significant differences in linear discriminant analysis (LDA) were found in the Ppca group and the Ctca group. LDA was used to compare the different relative abundance of bacterial taxa (LDA score threshold of 2.0). The red arrow (LDA score threshold of 2.0) indicates that the abundance of the bacterial genera increased in the Ppca group, and the green arrow (LDA score threshold of 2.0) indicates that the abundance of the bacterial genera decreased in the Ppca group.

Linear discriminant analysis (LDA) was used to compare the different relative abundance of bacterial taxa. LDA (score threshold, 2.0) revealed that 37 genera of bacteria in the intestines of housefly larvae were significantly different in abundance after continuous phage treatment (Fig. 8). Bacterial genera with large changes in abundance and the changes in their proportions were investigated. The proportion of *Lactococcus* (0.37% in the Ppca group and 0.13% in the Ctca group) increased significantly, while the abundance of *Helicobacter* (0.02% in the Ppca group and 0.33% in the Ctca group) decreased on the first day. On the second day of treatment, the proportion of Klebsiella (49.46%) in the Ppca group increased significantly (32.09% in the Ctca group), while the proportion of *Providencia* (43.75%) in the Ppca group decreased significantly (Ctca group, 55.40%). The proportion of Proteus (5.95% in the Ppca group and 0.68% in the Ctca group) increased significantly, while the abundance of *Bordetella* (0.01% in the Ppca group and 3.89% in the Ctca group) decreased on the third day. On the fourth day of treatment, the proportion of Klebsiella (30.90%) in the Ppca group increased significantly (2.30% in the Ctca group), while the proportion of *Providencia* (46.44%) in the Ppca group decreased significantly (Ctca group, 87.28%). Therefore, we speculate that, besides the decrease in Pseudomonas aeruginosa, the change in the proportion of dominant bacteria, such as *Providencia*, Klebsiella, Proteus, and *Bordetella*, in the intestinal tract of housefly larvae is another key factor affecting the health of houseflies.

**FIG 9:**
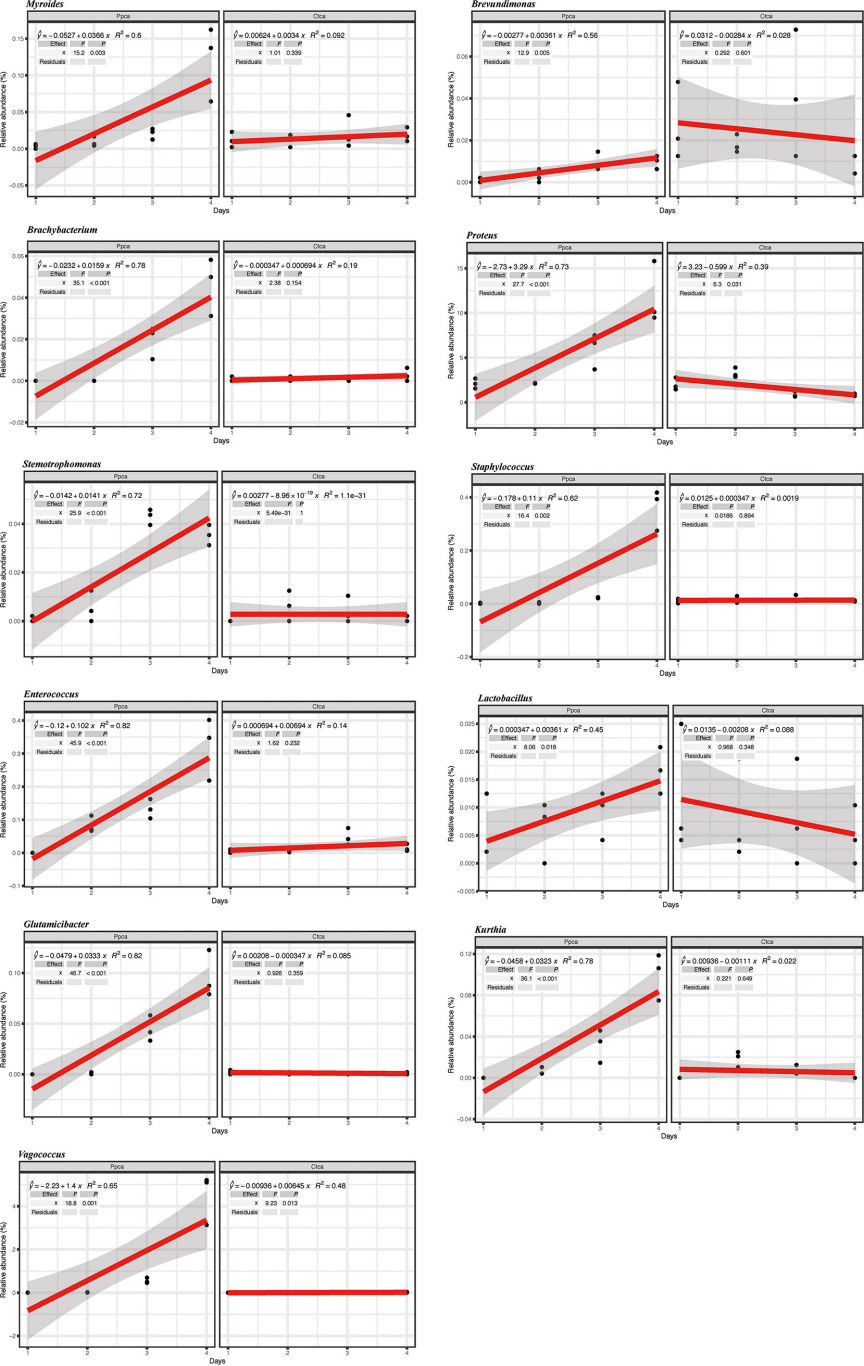
Positive correlations between the relative abundances of different bacterial genera and phage presence/absence in the experiment. The *R*^2^ and *P* values refer to the most parsimonious model fitted based on linear regression analysis.

**FIG 10:**
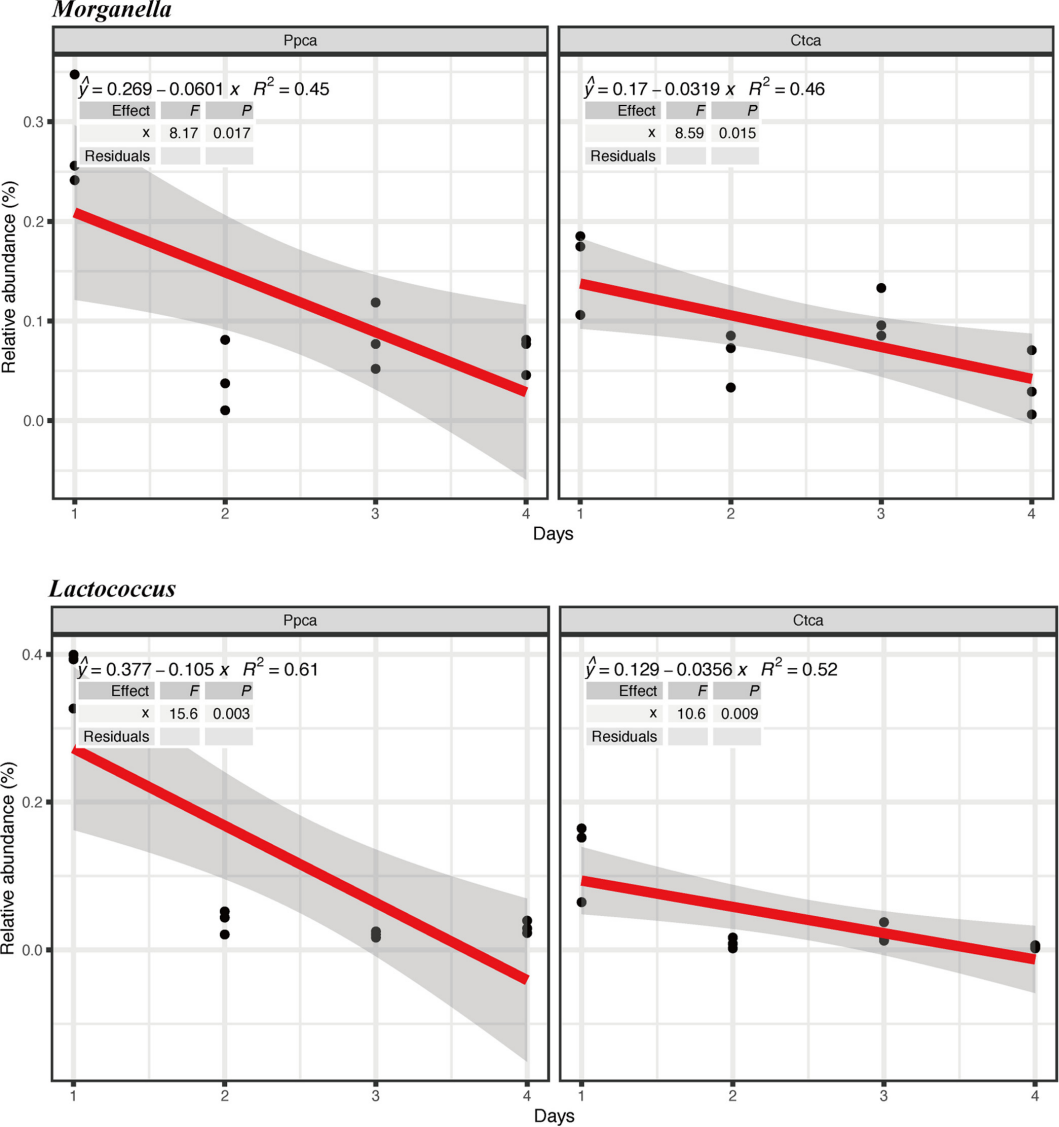
Negative correlations between the relative abundances of different bacterial genera and phage presence/absence in the experiment. The *R*^2^ and *P* values refer to the most parsimonious model fitted based on linear regression analysis.

Next, we found that some relative abundances of bacterial genera correlated mostly positively with the decrease in Pseudomonas aeruginosa in the community. Specifically, the decrease in Pseudomonas aeruginosa correlated with high relative abundances of *Brachybacterium* (*R*^2^ = 0.78, *P < *0.001), *Brevundimonas* (*R*^2^ = 0.56, *P = *0.005), *Enterococcus* (*R*^2^ = 0.82, *P < *0.001), *Glutamicibacter* (*R*^2^ = 0.82, *P < *0.001), *Lactobacillus* (*R*^2^ = 0.45, *P = *0.018), *Myroides* (*R*^2^ = 0.6, *P = *0.003), Staphylococcus (*R*^2^ = 0.002, *P = *0.002), *Stenotrophomonas* (*R*^2^ = 0.72, *P < *0.001), *Vagococcus* (*R*^2^ = 0.65, *P = *0.001), Proteus (*R*^2^ = 0.73, *P < *0.001), and *Kurthia* (*R*^2^ = 0.78, *P < *0.001) genera (Fig. 9). Although there was a negative correlation between *Morganella* and *Lactococcus* and the decrease in Pseudomonas aeruginosa, their abundances in the first 2 days were higher than those of the control group (Fig. 10). To determine the interactions between the cultivable bacteria and Pseudomonas aeruginosa, we conducted an antagonism assay. The antagonism assay revealed that the growth of Pseudomonas aeruginosa was inhibited by high concentrations of Lactococcus lactis, and the growth of Lactococcus lactis was also inhibited by Pseudomonas aeruginosa at high abundances (Fig. S6). These results suggest that these bacteria potentially contributed to the suppression of Pseudomonas aeruginosa via either resource or interference competition in intestines treated with phage.

**FIG 11:**
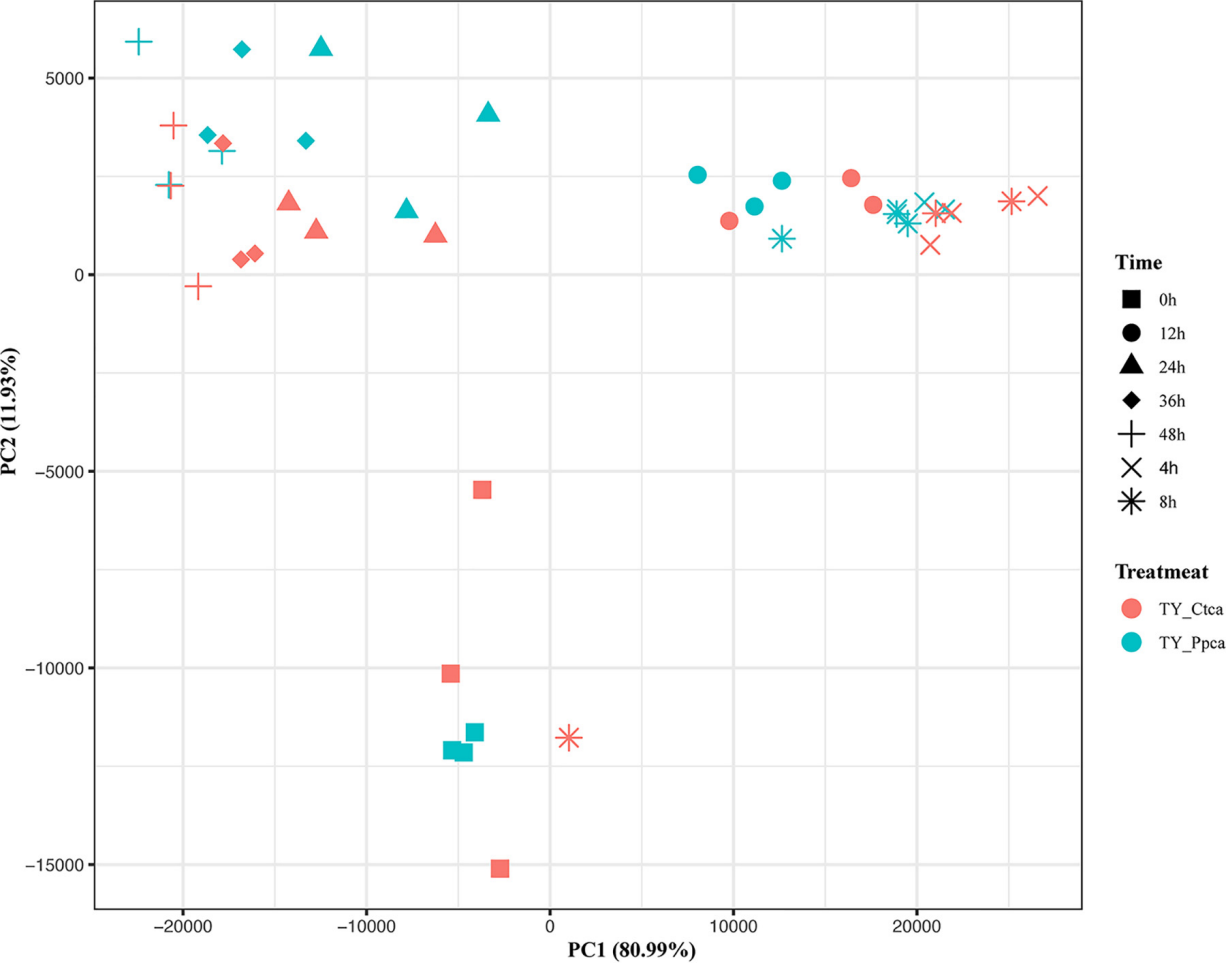
Differences in bacterial community structures and relationships between the two groups in *in vitro* experiments. TY-Ctca represents the *in vitro* experimental control group, and TY-Ppca represents the group receiving phage treatments every 24 h *in vitro*.

**FIG 12:**
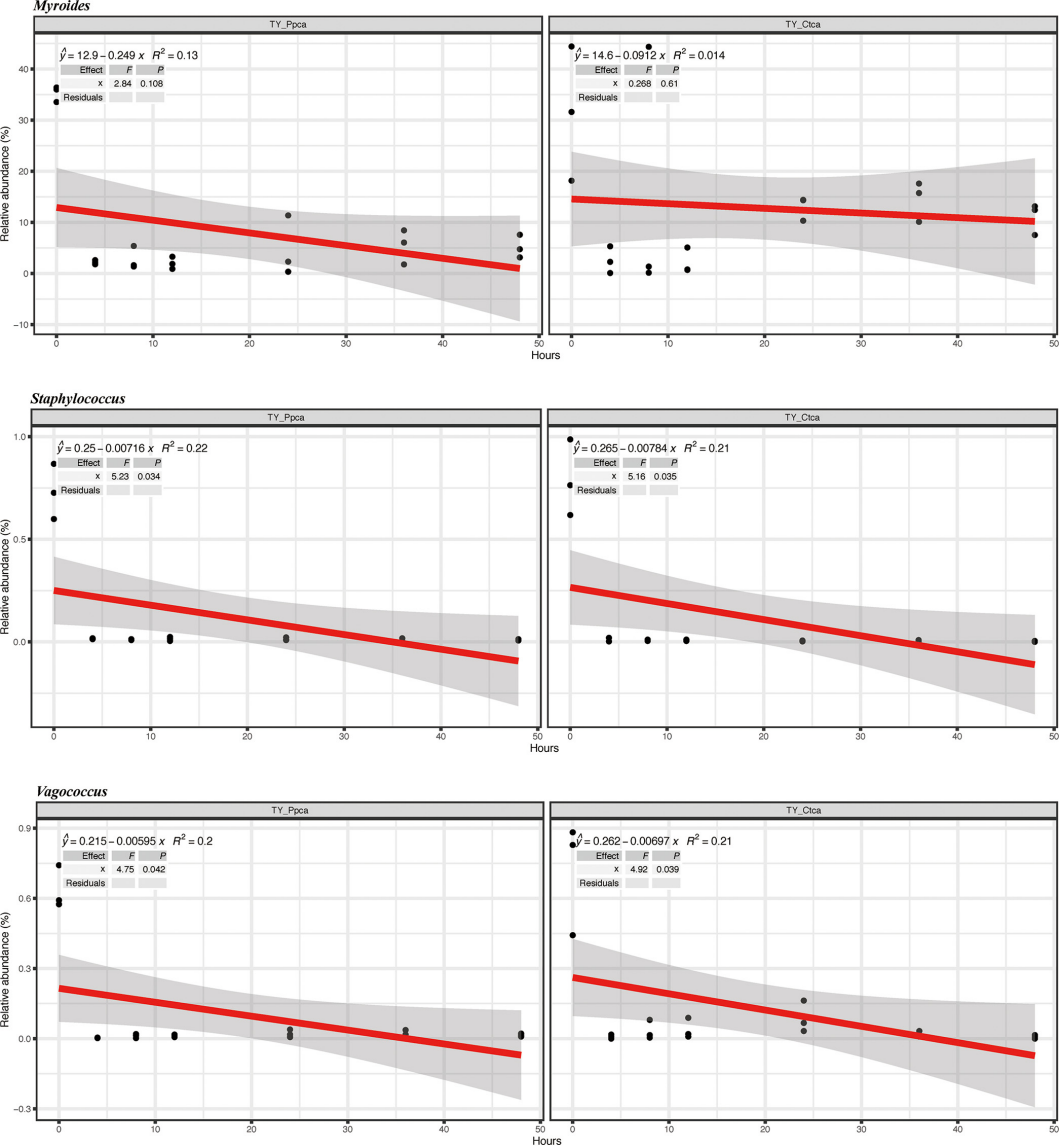
Bacterial genera with positive correlations in *in vivo* experiments lose their positive correlations. TY-Ctca represents the *in vitro* experimental control group, and the TY-Ppca represents the group receiving phage treatments every 24 h *in vitro*. The *R*^2^ and *P* values refer to the most parsimonious model fitted based on linear regression analysis.

To verify that these competitions were carried out in the gut environment, we used a short-term *in vitro* bacterial culture experiment to test how phages affected the composition and diversity of the intestinal community. We found that the composition of the microbiome did not change significantly with the presence of phage (Fig. 11). Moreover, *in vitro* experiments did not reveal a significant correlation between the Staphylococcus, *Myroides*, and *Vagococcus* genera (Fig. 12). These results show that the interactions of intestinal bacteria are inseparable from the intestinal environment.

### Bacterial networks in different housefly larvae.

**FIG 13:**
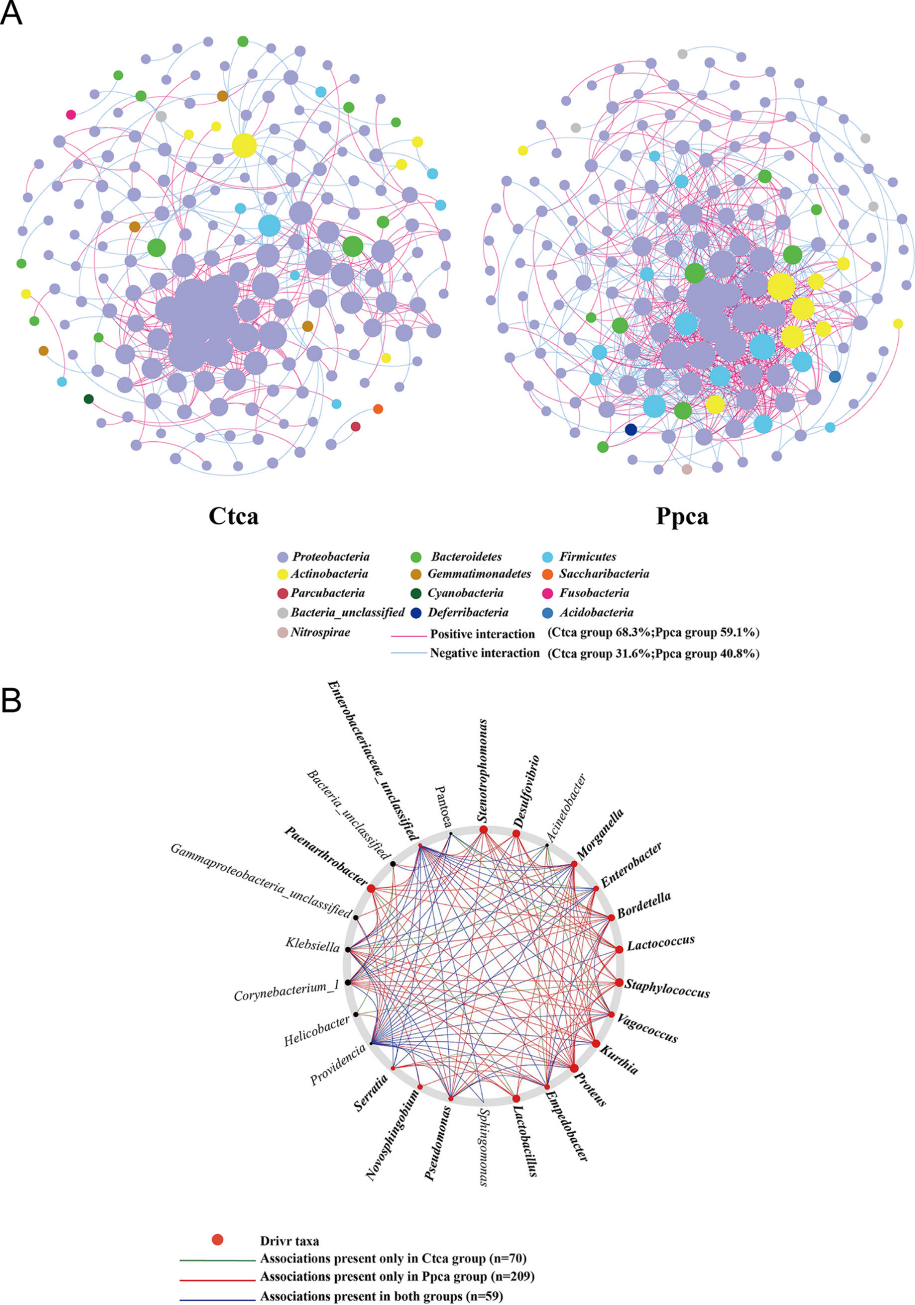
Intestinal bacterial co-occurrence microbiome networks of the Ctca and Ppca groups. (A) Effects of single phages on bacterial co-occurrence networks. Bacterial co-occurrence networks associated with single-phase experiments. Each node represents a bacterial OTU, and each edge represents a negative (displayed in blue) or positive correlation (displayed in red). The node colors represent taxon classifications at the phylum level. (B) Based on a NetShift analysis of the Ctca and Ppca groups, the potential driver taxa are essential for observing the correlated network co-occurrence bacterial changes after phage treatment and are marked Ppca-Ctca. Each node size is proportional to its neighbor shift (NESH) score (a score identifying the importance of a given microbial taxon in the association network), and the nodes colored red are important driver taxa. As a result, the large red nodes represent driving taxa that are particularly important during phage infection. Line colors indicate node (taxa) connections: the association only appears in the case groups (red edge), the association only appears in the control group (green edge), and the association is in both the case groups and the control group (blue edge).

In this study, to analyze the interactions within bacterial communities in more detail, we constructed co-occurrence networks based on OTUs. The positive and negative relationships between gut bacterial communities were analyzed after a single phage was added to inhibit Pseudomonas aeruginosa. NetShift analysis was used to identify potentially important taxa driving community change (41). After initial screening, the Ctca group (68.3% positive versus 31.6% negative significant correlations) and Ppca group (59.1% positive versus 40.8% negative significant correlations) co-occurrence networks included 183 and 166 nodes, respectively, and 405 and 778 edges with clustering coefficients of 0.186 and 0.249, respectively (Fig. 13A; Table S3). The decrease in Pseudomonas aeruginosa increased the negative correlations between the flora; that is, the competition between the bacteria increased. This is consistent with some of the aforementioned bacteria that grow with positive correlations. The co-occurrence networks of the Ctca group were initially much larger than those of the Ppca group, having more nodes, a longer average path distance, and larger maximal betweenness, which proved that the housefly larvae of the control group were healthier (42) than those of the Ppca group (Fig. 13A). On average, phage group networks were more connected, with the largest number of edges, the largest clustering coefficient, and the shortest average path length, indicating that there are potentially more frequent interactions between the bacteria (Fig. 13A). Moreover, the co-occurrence network diagram shows that the presence of phages strengthens the interactions between *Firmicutes*, *Actinobacteria*, and *Proteobacteria*. Therefore, the removal of a single bacterium in the intestine strengthens the interactions of other bacterial groups. It is speculated that this is a key factor in intestinal disorders.

NetShift analysis showed that most of the taxon associations were completely different between Ppca group and Ctca group communities and that the number of significant associations increased with the presence of phage (Fig. 13B; 70 and 209 associations, respectively). NetShift analysis revealed 26 (Ppca-Ctca) potential driver taxa. Among these driver taxa, we considered *Bordetella*, *Morganella*, *Stenotrophomonas*, *Kurthia*, *Desulfovibrio*, Staphylococcus, *Vagococcus*, Enterobacter, Proteus, *Lactococcus*, *Empedobacter*, *Lactobacillus*, Pseudomonas, *Serratia*, *Novosphingobium*, *Paenarthrobacter*, and *Enterobacteriaceae*_unclassifieds the key bacterial genera in the Pp group after the removal of Pseudomonas aeruginosa. These genera were found to play important roles in changing the network structure of the Ppca group.

## DISCUSSION

In this study, we show that the health of housefly larvae was negatively affected by the phage-mediated reduction in Pseudomonas aeruginosa. The removal of certain bacteria is mediated by bacteriophages, which can be used as a precision tool to regulate the density of certain bacteria in the insect gut. Notably, the continuous targeting of bacteria by bacteriophages indirectly changes the composition of intestinal microbes and the bacterial symbiosis network. Our results revealed that the negative effects caused by the phage-mediated loss of certain bacteria should not be neglected when phages are used as a precision tool to precisely regulate the composition of the insect intestinal microbiota and to study bacteria-insect-intestinal microbiota interactions.

Continuous administration of phages was sufficient to suppress bacterial growth in the gut of housefly larvae. However, the targeted bacteria became resistant to subsequent phages. These results are consistent with those of previous studies (38) showing that bacteriophages can significantly inhibit the growth of intestinal bacteria. Previous studies have also shown that increasing the amount of phages improved the biocontrol efficacy of phage combinations (43, 44). Phages in phage combinations kill pathogens through different receptors or mechanisms, which limits the evolution of bacterial resistance in phage therapy (45, 46). Only one type of phage was used in our experiment, after which the bacteria quickly rebounded and became resistant to subsequent phage treatment. It is important to study the effects of multiple phage combinations on the removal of insect intestinal bacteria in the future.

Moreover, we show that bacteriophages can specifically target Pseudomonas aeruginosa, which provides a method for precisely regulating the bacterial load of certain bacteria in the intestinal microbiota. Antibiotics are often used in sterile animal models to remove all bacteria in the intestinal tract of insects. Our previous research revealed that low-concentration P. aeruginosa Y12 could protect housefly larvae from entomopathogenic fungal infection through the production of antifungal metabolites. According to our previous research, application of strain Y12 and antibiotics induced the highest mortality during B. bassiana infections compared to the control group. However, broad-spectrum antibiotics could remove most Gram-negative bacteria without specificity. In this research, we aim to use the specificity of bacteriophages that target some bacteria while not negatively affecting other microbiota to study the interactions between different bacteria in the intestinal flora. There are significant differences in the growth and development of germ-free insects and insects with one or several bacterial strains removed (47); when sufficient Acetobacter pomorum (a symbiotic bacterium isolated from the digestive tract of insects) colonizes, the insects can restore the development and growth of larvae (48). However, the lack of all bacteria inevitably leads to insect diseases, and the addition of any kind of bacteria may restore health. This makes it impossible to judge the effects of a single bacterium in the presence of the rest of the microbiota. Moreover, it is not clear how an extreme lack of a single symbiotic bacterium affects the life activities of insects and the composition of the gut microbiota under the conditions of normal colonization of insect intestinal bacteria. Therefore, this motivated us to search for organisms or agents that target specific bacteria. Our results indicate that the use of bacteriophages to reduce the amount of Pseudomonas aeruginosa in the intestines of housefly larvae has a negative impact on the growth and development of housefly larvae. Two main factors would explain why continuous bacteriophage treatment would result in developmental delays in housefly larvae. The first is that the bacteriophage-mediated lysis of Pseudomonas aeruginosa releases multiple bacterial compounds that could be harmful to housefly larvae, while the second reason is that the reduction in phage-targeted Pseudomonas aeruginosa disturbs the balance of the intestinal flora, which further affects the development of housefly larvae. The latter reason has been noted by multiple investigators, and it has yet to be directly tested *in vivo*. We assume that the interactions between bacteriophages and bacteria change the interactions between the bacteria and insects, thereby affecting the growth and development of insects. This kind of ecological evolutionary feedback has been found in various other systems (49–51). Our results show that it is important to study phage-intestinal bacteria-insect interactions in the intestinal environment.

The interaction between bacteria and bacteriophages in the natural environment fully demonstrates the ability of bacteriophages to shape gut microbial colonies (52, 53). However, there are few reports discussing when and how phages shape the abundances and compositions of host-associated bacteria in the gut. The experiments targeting Pseudomonas aeruginosa with phage in our research investigated the changes in the community composition and co-occurrence network of intestinal microbes. These changes were indirect, as the phage did not infect bacteria other than Pseudomonas aeruginosa. Notably, we found that the relative abundances of *Enterococcus*, Proteus, *Vagococcus*, *Glutamicibacter*, and *Kurthia* positively correlated with the presence of phage. This shows that the density of Pseudomonas aeruginosa is negatively correlated with the density of these bacteria. We assume that the decrease in Pseudomonas aeruginosa increases the habitable space and available nutrients for other microorganisms and that they have a strong antagonistic effect on the bacteria targeted by the phage through resource competition or interference competition (54, 55). Our competition experiment of incubating cultivable bacteria and Pseudomonas aeruginosa in a microaerobic environment also fully verified this competition mechanism. In addition, *in vitro* experiments have fully confirmed that the intestinal environment is a necessary condition for the interactions in the microbiota. Moreover, we identified several candidate driver taxa that played a key role in bacterial co-occurrence networks. For example, the *Serratia* genus has previously been shown to be pathogenic to various insects, promoting the death of *Anopheles* (56) and increasing the mortality of the cotton leafworm Spodoptera littoralis (57). It is thus possible that a lack of symbiotic bacteria leads to increased interactions with harmful bacteria, which is also a key factor affecting insect health. Therefore, bacteriophages are essential in the maintenance of the intestinal flora balance in housefly larvae and play a key role in the study of insect-microbiota interactions.

In conclusion, we found that phage-mediated loss of certain gut bacteria has a significant impact on insect health and the composition of gut bacteria. The use of bacteriophages in the insect gut model showed promise in the future for regulating the composition of the gut microbiota. To date, the application of bacteriophages in insect models is still rarely reported. Our research is the first to explore the dynamic changes in the intestinal microbiota at various stages when phages invade the intestines of housefly larvae. These findings provide new insights into the health effects of the interactions between insect gut bacteria on insects when bacteriophages are used as sophisticated tools for regulating the composition of intestinal microbes.

## MATERIALS AND METHODS

### Microbial strains and experimental conditions.

We used a Pseudomonas aeruginosa strain isolated from the intestines of housefly larvae as a host bacterium for screening phages in our experiments. The housefly larval strain was reared in the vector biological laboratory of Shandong First Medical University for approximately 15 years without exposure to pesticides or entomopathogenic fungi. A lytic phage of Pseudomonas aeruginosa was isolated from a river in Tai’an, China. This phage, named Y12Pw, was used in our phage infection experiments (Table S1 in the supplemental material). Y12Pw showed a lytic nature by causing clear lysis of the stock Pseudomonas aeruginosa strain on double-layer agar plates (Fig. 1A). Phages were isolated by the double-layer plate method (58) and were purified by constantly picking individual plaques (59). Picking a single phage spot on a double-layered agar plate was repeated until the plaques were homogeneous, and phages were then morphologically identified by transmission electron microscopy. Briefly, the initial phage stocks were prepared by mixing the phage and Pseudomonas aeruginosa strain cultures in LB broth (yeast extract 5.0 g liter^−1^, tryptone 10.0 g liter^−1^, NaCl 10.0 g liter^−1^) and incubating for 24 h, with the addition of centrifugation (5 min at 8,000 × *g*) and filtration (0.22 μm) steps to isolate and purify the phage from the bacterial cells. Subsequently, polyethylene glycol (PEG) precipitation was used to enrich the phage particles. The phage titers were adjusted to 10^8^ phage particles per ml, and phage stocks were stored at 4°C.

### Electron microscopy.

Negative stain transmission electron microscopy was used to image purified phage preparations. The purified phage (10^9^ PFU/ml) was placed on a carbon-coated copper grid, washed with deionized water, stained with 1% uranyl acetate for 20 s, and subsequently air dried. The specimen was blotted with filter paper between steps.

### Phage sequencing.

Phage chromosomal DNA was isolated using the λ phage genomic DNA purification kit (ABigen) following the manufacturer’s instructions. Whole-genome sequencing was performed with an Illumina HiSeq 4000 platform. The METAVIRALSPADES pipeline (60) was used to identify the phage in the sample (mainly including sequence assembly, phage sequence identification, and phage integrity identification). The genome of Y12Pw has 282,378 bp with an average GC content of 36.82%, and the phage was assigned to the *Caudovirales* order and *Podoviridae* family based on morphology and sequence similarity with the other phages (Fig. 1 and 2). Gene predictions and annotations were performed using GeneMarkS. For the single protein-based phylogenetic analysis, a neighbor-joining phylogenetic tree based on phage tail fiber protein similarity between the phage Y12Pw used in this experiment and other 10 similar phages that are publicly available at NCBI was generated (Fig. 2). Phylogenetic trees, based on 500 bootstrap replicates, were constructed by using neighbor-joining (NJ) methods using MEGA 6.0 (61). A genome-based phylogenetic tree of Y12Pw and the other 72 phages (Fig. 3A) that belong to *Myoviridae*, downloaded from NCBI, was constructed. Briefly, MAFFT (v 7.471) (62) was used to align the multiple genome sequences under its default options. Then, the maximum likelihood phylogenetic tree was constructed by FastTree (v2.1.11) based on the aligned genome sequences. The final phylogenetic tree was explored using the FigTree software (v1.4.4) (63).

### Assessment of the bactericidal efficacy of the phage in the intestines of housefly larvae and collection of intestine samples at the end of the experiment.

We selected 1-day-old housefly larvae hatched for a 4-day feeding experiment to test the bactericidal efficacy of phage in the presence of a natural intestinal microbiome. The diet of housefly larvae was composed of sterilized wheat bran and sterilized water (1:1) and was mixed into a paste. The sterile water of the experimental group contained ∼10^8^ total phage particles per ml. The control treatment did not include the addition of phages. The phage treatment group regularly received ∼10^8^ phage particles per ml every day. They were reared in a 5-ml perforated test tube to ensure ventilation and were placed in an incubator with a temperature of 26 ± 1°C, a relative humidity of 60% ± 10%, and an illumination of 12:12 (light:dark). The experiment in each group was performed in three perforated test tubes independently, and four samples of each test tube were sampled every day as replicates. The body length and weight of housefly larvae were regularly recorded every day. The pupation rate and emergence rate were recorded at the end of the experiment. The housefly larvae in different test tubes were collected daily until 4 days after treatment. The surfaces of housefly larval samples were thoroughly cleaned with sterile water, and then the whole intestine was dissected under sterile conditions. The dissected intestine was washed with sterile water three times and then placed in sterile centrifuge tubes containing sterile normal saline, with one sample in each tube, and the sample number was marked. The washing liquid was collected and filtered to determine the titer of the intestinal phage of each group of housefly larvae. The double-layer agar plate method (58) was used to count phage plaques for the determination of phage titer. The intestinal bacteria solution was used to determine the amount of Pseudomonas aeruginosa in the intestine. Pseudomonas aeruginosa selection medium (CHROMagar, PS830) was used for isolation and detection of intestinal Pseudomonas species, including P. aeruginosa. The chromogenic medium enables detection of colonies of P. aeruginosa by their blue color, while other microorganisms, such as Staphylococcus saprophyticus, Escherichia coli, and Proteus mirabilis, would be inhibited and colorless. Finally, 10 intestinal samples were mixed together as a sample unit. Each sample unit was a pool containing 10 intestines for phage-infected housefly larvae or controls. Three units of 10 repetitions were used for 16S rRNA high-throughput sequencing of intestinal bacteria. The 16S sequencing data of the microbiome were deposited in the SRA database and can be accessed by the BioProject accession number PRJNA746668.

### Quantification of phage resistance with evolved P. aeruginosa isolates.

To determine the evolution of phage resistance during every 24 h, we performed an *in vitro* experiment to check the development of resistance against the bacteriophage with one addition and multiple additions every 24 h. Twelve colonies were set up to determine phage resistance. Briefly, the growth of ancestral and evolved P. aeruginosa bacterial colonies (inoculum of ∼10^8^ cells per ml) were measured both in the absence and presence of each ancestral phage population (inoculum of ∼10^8^ phage particles per ml) on 96-well microplates at 30°C using a spectrophotometer at an 8-h time point (optical density at 600 nm [OD_600_]) (29).

### Testing the direct effects of phage on bacterial community composition and diversity using culture-dependent and culture-independent methods.

We used a culture-independent method to directly test whether phage-mediated deletion of Pseudomonas aeruginosa caused competition with other intestinal bacteria in the absence of an intestinal environment. Briefly, 1 to 5 instar housefly larva intestinal microbe milling liquid was cultured in 60 ml of LB liquid medium for 48 h. A control group and a phage treatment group, each with three parallel samples, were set up. In the phage treatment group, ∼10^8^ phage particles per ml was added every 24 h. We sampled the bacteria in the flask at 0 (without any treatment, but the phage was added immediately after sampling), 4, 8, 12, 24, 36, and 48 h. After washing the bacterial solution three times, the bacteria were obtained and placed in a sterile test tube for storage. Three replicate samples for each period in each group were used for 16S rRNA high-throughput sequencing of bacteria cultured outside the intestinal environment.

### Plate confrontation assay of Pseudomonas aeruginosa and Lactococcus lactis in a microaerobic environment.

To determine the interactions between the cultivable bacteria and Pseudomonas aeruginosa, we conducted plate confrontation experiments in nutrient agar (NA) medium plates (peptone 10.0 g liter^−1^, agar 20 g liter^−1^, NaCl 5.0 g liter^−1^, beef extract 3.0 g liter^−1^) in a microaerobic environment. P. aeruginosa and Lactococcus lactis were inoculated in LB liquid medium (yeast extract 5.0 g liter^−1^, tryptone 10.0 g liter^−1^, NaCl 10.0 g liter^−1^) and cultured at 37°C overnight (OD_600_ of >1.0). P. aeruginosa and Lactococcus lactis cultures were inoculated on half of a nutrient agar plate using the spread plate method with a sterile cotton swab, and the opposite side of the agar plate was used as a negative control. On the P. aeruginosa plate, two 6-mm-diameter sterile filter papers were dipped in the Lactococcus lactis bacterial liquid. After slight drying, the filter papers were placed opposite on the two sides of agar medium. On the Lactococcus lactis spread plate, the two 6-mm-diameter sterile filter papers were dipped in P. aeruginosa bacterial liquid with subsequent experiments performed as described above. All plates were incubated at 37°C and placed in an anaerobic incubator for 24 h. Finally, the bacterial growth diameter was recorded. The colony sizes of P. aeruginosa and Lactococcus lactis were measured to evaluate the interactions. The experiments were conducted with six independent biological replications.

### DNA extraction of the intestinal bacteria.

The intestinal samples were homogenized in a tissue lyser (Qiagen, Hilden, Germany) followed by genomic DNA isolation using the Wizard genomic DNA purification kit (Promega, A1120), according to the manufacturer’s instructions, with DNA suspended in 30 μl of nuclease-free water. The concentration and quality of extracted DNA were assessed using a NanoDrop 2000 spectrophotometer (Thermo Fisher Scientific, Waltham, MA, USA) and 2% agarose gel electrophoresis, respectively. Extracted DNA was stored at −20°C until further processing.

### Illumina sequencing and bioinformatics analysis.

The hypervariable V3-V4 region of the bacterial 16S rRNA gene was amplified with the primers 341F (5′-CCTAYGGGRBGCASCAG-3′) and 806R (5′-GGACTACNNGGGTATCTAAT-3′). Twenty-microliter PCR mixtures were set up with 4 μl of 5× FastPfu buffer, 2 μl of deoxynucleoside triphosphates (dNTPs) (2.5 mM), 0.8 μl of each primer, 0.4 μl of FastPfu polymerase, and 10 ng of template DNA. Reactions proceeded in a GeneAmp 9700 (ABI) thermocycler with the following parameters: 95°C for 5 min, 27 cycles of denaturation at 95°C for 30 s, annealing at 55°C for 30 s, and elongation at 72°C for 45 s, followed by additional elongation at 72°C for 10 min and a dissociation stage at the end of the run.

PCR products were detected by 2% agarose gel electrophoresis and were purified using the QIAquick gel extraction kit (Qiagen). Library pools were constructed with equal amounts of each PCR product by using the TruSeq Nano DNA LT sample prep kit (Illumina), which was amplified through paired-end sequencing on the Illumina MiSeq PE300 platform.

Quality control of the original data was performed using Trimmomatic v0.39 software (http://www.usadellab.org/cms/index.php?page=trimmomatic). Based on the overlap (minimum: 10 bp) between paired-end (PE) reads after quality control, PE reads were assembled using FLASH v1.2.11 software (fast length adjustment of short reads to improve genome assemblies). QIIME v1.9.1 software (allows analysis of high-throughput community sequencing data) was adopted for processing, and VSEARCH v2.14.1 software (a versatile open-source tool for metagenomics) was used for detecting chimeric sequences. Based on a sequence similarity level of 97%, the UCLUST method in QIIME software was employed to perform OTU clustering analysis. On the basis of the Silva reference database (release 138), taxonomic annotations were made for the OTUs in each sample. The Shannon, Simpson, Chao1, and Ace indexes of microbial communities were calculated by mothur (https://mothur.org/). LEfSe software (http://huttenhower.sph.harvard.edu/galaxy/) was used to estimate the abundance differences among microbial species in samples.

Principal coordinate analysis (PCoA) based on Bray-Curtis dissimilarity and an unweighted pair group method with arithmetic mean (UPGMA) tree based on unweighted UniFrac phylogenetic distances were used to determine the difference in bacterial community beta diversity in different samples. Bray-Curtis ordination is an effective strategy for the analysis of multivariate ecological data. Fast UniFrac facilitates high-throughput phylogenetic analyses of microbial communities, including analysis of pyrosequencing and PhyloChip data.

### Statistical analysis.

Paired-end reads were assigned to samples based on their unique barcodes and were truncated by cutting off the barcode and primer sequence. Then, the paired-end reads were merged into longer single sequences using FLASH (v1.2.11) (64). Quality filtering was performed on the raw tags under specific filtering conditions to obtain high-quality clean tags (65) according to the QIIME (v1.8.0) (66) quality control process. OTUs were clustered with a 97% similarity cutoff using VSEARCH (v2.15.0). Chimeric sequences were detected and removed using UCHIME (v4.2.40) (67). Representative sequences from each OTU were screened for further annotation. For each representative sequence, the Silva database (release 138) was used with RDP Classifier (v2.2) (68) to obtain taxonomic information. Microbial diversity was analyzed using QIIME v1.8.0 and was displayed using R software (v3.0.3) (66). The alpha diversity analysis included observed species, Ace and Chao1 estimators, and the Simpson and Shannon diversity indexes. The mean and standard deviation values of variables were calculated using Microsoft Excel 2016. Before the analysis of variance (ANOVA), the homogeneity of the variances was visually verified by plotting the residuals against the predicted values. ANOVA was performed to determine the effects of phage infection on the body length and weight of house fly larvae and the number of P. aeruginosa in the intestines of the two groups of housefly larvae indexes using SAS software (69). Data were analyzed with a linear regression and were explained by the phage-mediated reduction of Pseudomonas aeruginosa relationship with the abundance of other bacteria genera. Networks were drawn using Gephi, and the ‘NetShift’ (42) method was used to identify potential keystone driver taxa underlying differences in microbiomes exposed to a single phage and no phage.

### Data availability.

The intestinal bacterial 16S rRNA gene sequences described here were submitted to BioProject, with ID number PRJNA746668, and the sequence of the Y12Pw phage was submitted to GenBank under accession number MZ444140.
