# Supplementary material for: Bacteriophage: A Useful Tool for Studying Gut Bacteria Function of Housefly Larvae, Musca domestica
Source: Microbiol Spectr. 2021 Aug 11;9(1):10.1128/spectrum.00599-21. doi: 10.1128/spectrum.00599-21 (PMC8552782; doi:10.1128/spectrum.00599-21)
Supplement: SUPPLEMENTAL FILE 1 — Supplemental material. Download SPECTRUM00599-21_Supp_1_seq1.pdf, PDF file, 2.3 MB [file spectrum00599-21_supp_1_seq1.pdf]

Bacteriophage: a useful tool for studying gut bacteria function of housefly larvae, musca domestica

Xinyu Zhang[1, 2#], Shumin Wang [2#], Ting Li[2], Qian Zhang[1, 2], Ruiling Zhang[1, 2*], Zhong Zhang[1, 2*]

[1] Collaborative Innovation Center for the Origin and Control of Emerging Infectious Diseases, Shandong First Medical University (Shandong Academy of Medical Sciences), No. 619, Changcheng Road, 271016 Taian, Shandong, China

[2] School of Basic Medical Science, Shandong First Medical University (Shandong Academy of Medical Sciences), Taian 271016, Shandong, China

*** Correspondence**：

Zhong Zhang, E-mail: nasonia@163.com

Ruiling Zhang, E-mail: rlzhang@tsmc.edu.cn

[#]These authors have contributed equally to this work.

Supplementary Tables:

Table S1 Table showing the name, location, year of isolation and morphology of the phage used in this study.

Name	Location and year of isolation	Morphology
Y12Pw	Taian China (36° 12' 26" E, 117° 6' 4" N) 2019	*Caudovirales (order)* *Myoviridae (family)*

Table S2 Information about 16S rRNA gene analysis in this study. Data are expressed as the mean ± standard deviation of three replicate samples in each sampling.

Samples		Clean Reads	Normalized reads	OTU number	Shannon	Simpson	Chao1	Coverage
**Ctca1**	**Ctca1_1**	53042	48059	360.33±84.68	1.39±0.04	0.4008±0.0274	735.33±166.63	0.9965±0.0006
	**Ctca1_2**	59119	48059					
	**Ctca1_3**	63412	48059					
**Ctca2**	**Ctca2_1**	62649	48059	458.00±66.02	1.14±0.86	0.3494±0.0194	819.33±215.22	0.9959±0.001
	**Ctca2 _2**	56124	48059					
	**Ctca2_3**	56916	48059					
**Ctca3**	**Ctca3_1**	64217	48059	529.67±6.66	1.40±0.21	0.441±0.1091	907.33±98.74	0.9957±0.0003
	**Ctca3_2**	64690	48059					
	**Ctca3_3**	60362	48059					
**Ctca4**	**Ctca4_1**	62619	48059	645.00±499.77	1.21±0.83	0.613±0.1984	1019.00±274.31	0.9962±0.0014
	**Ctca4_2**	74963	48059					
	**Ctca4_3**	72710	48059					
**Ppca1**	**Ptca1_1**	60247	48059	278.33±22.59	1.32±0.18	0.8739±0.8053	643.00±93.74	0.997±0.0002
	**Ptca2_2**	52065	48059					
	**Ptca1_3**	54258	48059					
**Ppca2**	**Ptca2_1**	72519	48059	453.00±183.28	1.24±0.08	0.4136±0.0097	833.33±263.21	0.9964±0.0018
	**Pca2 _2**	64404	48059					
	**Ptca2_3**	66846	48059					
**Ppca3**	**Ptca3_1**	63580	48059	412.00±20.66	1.43±0.15	0.3976±0.0755	865.67±91.54	0.9961±0.0006
	**Ptca3_2**	62619	48059					

	**Ppca3_3**	54303	48059					
	**Ppca4_1**	53875	48059					
**Ppca4**	**Ppca4_2**	55875	48059	392.00±16.52	1.80±0.07	0.2824±0.0188	744.00±54.34	0.9963±0.0003
	**Ppca4_3**	61849	48059					

Table S3 Topological properties of bacterial co-occurrence networks associated with no-phage and single-phage treatments.

Group	Network index	Total nodes	Total links	Average clustering coefficient	Average path distance	Maximal betweenness
**Ctca**	0.820	183	405	0.186	5.074	3996.842
**Ppca**	0.740	166	778	0.249	3.397	2073.992

Table S4 Information on all the driver taxa associated with bacterial communities exposed to phage and not exposed to phage based on network analysis.

Genera	Jaccard Index	NESH score	Delta-betweenness	Degree in Ctca	Degree in PPca	Degree in both
Bordetella	0.25	1.845	0.041	5	15	4
Morganella	0.368	1.708	0.143	7	19	7
Stenotrophomonas	0.091	2.189	0.007	1	11	1
Kurthia	0.143	2.159	0.028	2	13	2
Desulfovibrio	0.111	2.074	0.006	1	9	1
Staphylococcus	0	2.279	0.001	1	10	0
Vagococcus	0.286	1.69	0.0017	5	13	4
Enterobacter	0.308	1.49	0.013	6	11	4
Proteus	0.167	2.315	0.072	2	19	2
Lactococcus	0.083	2.12	0.012	2	11	1
Empedobacter	0.438	1.458	0.068	7	16	6
Lactobacillus	0	2.079	0.003	1	6	0
Pseudomonas	0.429	1.331	0.016	8	13	6
Serratia	0.5	1.148	0.012	4	8	4
Novosphingobium	0.333	1.407	0.001	1	3	1
Paenarthrobacter	0	2.233	0.006	1	8	0
Enterobacteriaceae_unclassified	0.652	0.992	0.069	16	24	16

Supplementary Figures:

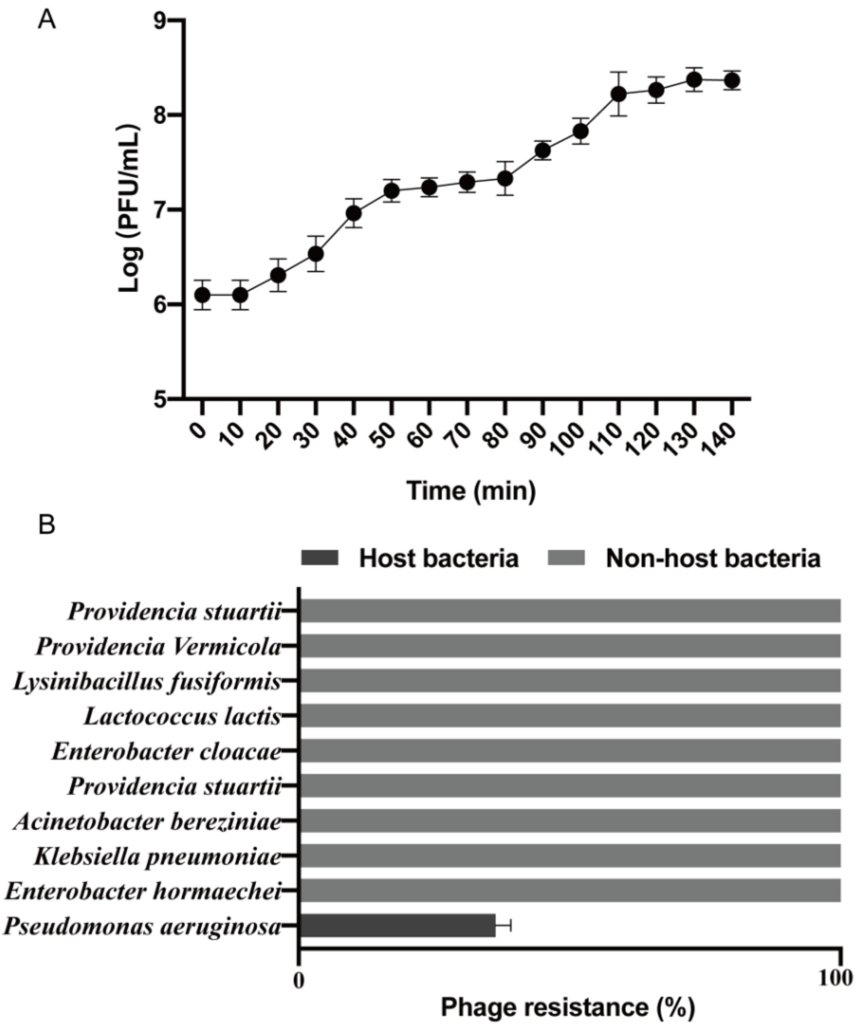

Figure S1 Biological characteristics of phage Y12Pw. (A) One-step growth curve of the phage Y12Pw. The Y-axis shows the log of plaque forming units per milliliter (PFU/mL). Experiments were repeated three times with duplicate samples. **(B) The resistance of *Pseudomonas aeruginosa* and other cultivable bacterial isolates in the housefly larval intestine to phage used in the experiments.** The infectivity of Y12Pw phage against host bacteria non-host bacterial isolates (other cultivable bacteria) from the housefly larval intestine.

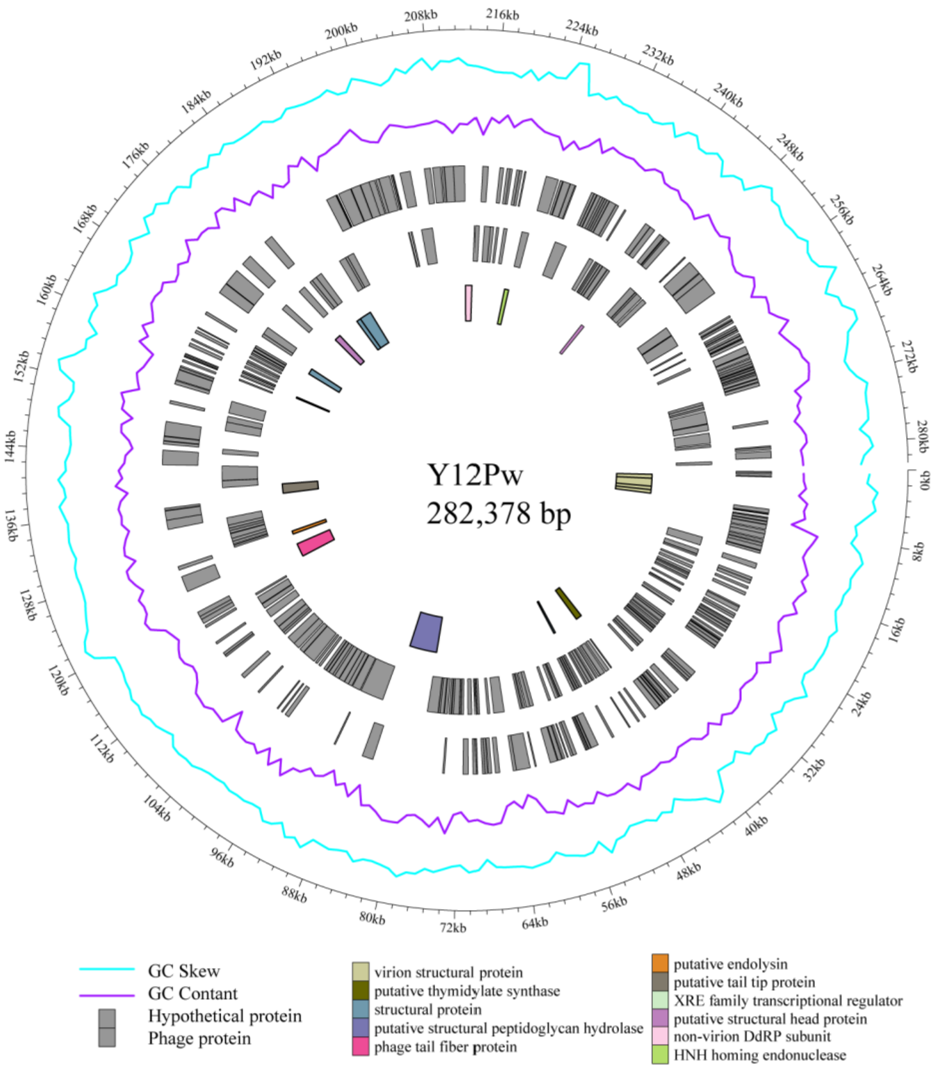

Figure S2 Annotated genome maps for the phage Y12Pw. In the circular genome map, the outermost black circle represents the full length of the genome, the innermost multicolored circle represents annotated functional proteins, the second outermost blue circle represents the GC skew, and the third outermost purple circle represents the GC skew content.

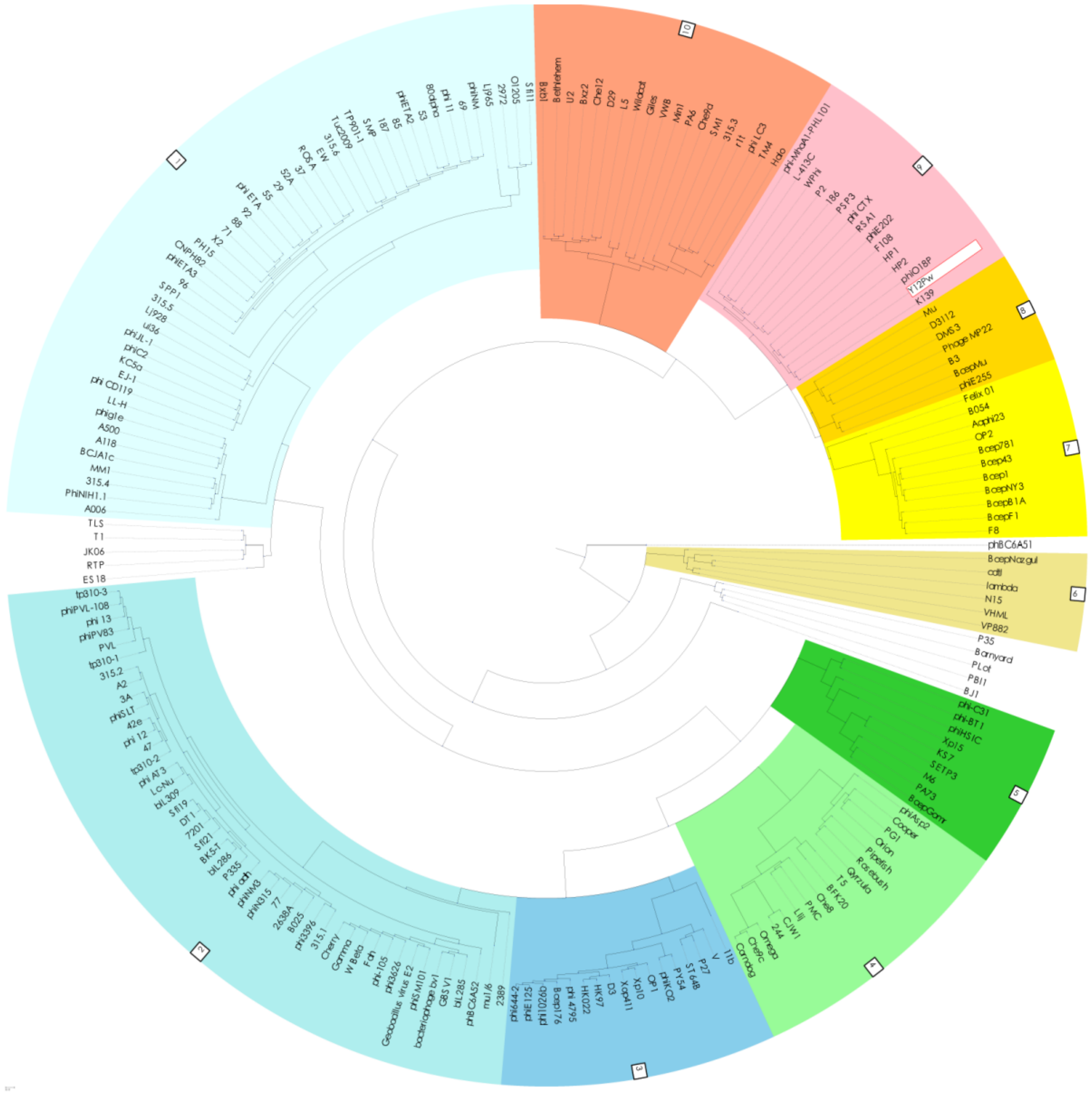

Figure S3 VIRFAM analysis is used to the recognition of head-neck-tail modules.

a clustering of the query phage with respect to those of same Type in the Aclame database. The head-neck-tail modules protein of Y12Pw (in text box with red border and white background) is similar with K139 (K139 belongs to the *Mycoviridae* family).

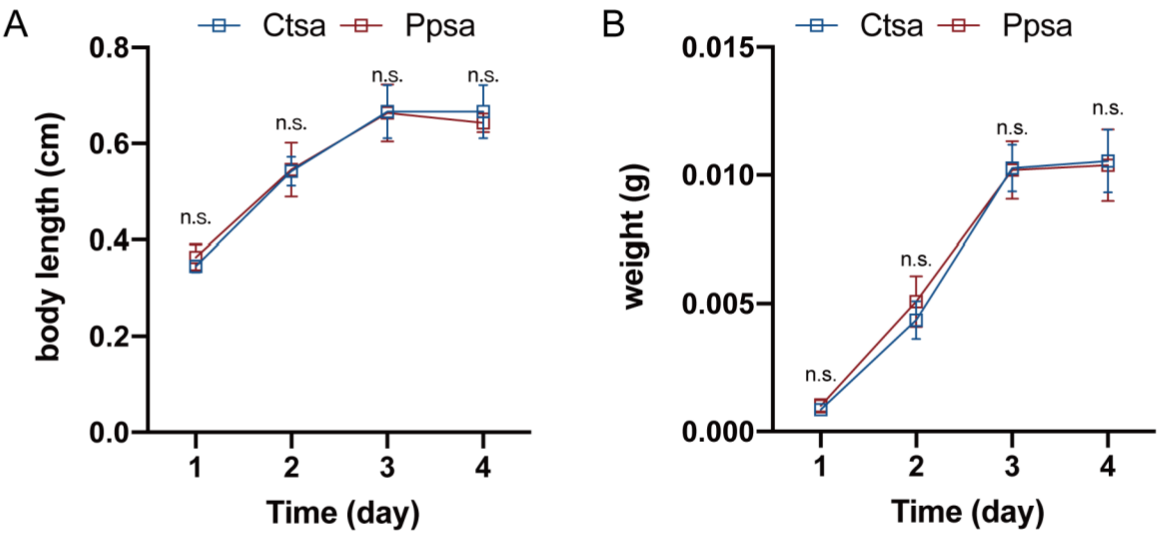

Figure S4 Changes in body weights and body lengths of housefly larvae treated with sterile water and sterile water containing less than 10^8 PFU/mL bacteriophage. (A) Body weight and body length (B) changed over time in housefly larvae with different treatments. Ctsa and Ppsa represent housefly larvae samples treated with sterile water and sterile water containing less than 108 PFU/mL bacteriophage, respectively. Data are shown as the mean ± SEM. Each treatment included 12 biological replicates. Repeated measures ANOVA was followed by Sidak correction for multiple comparisons.

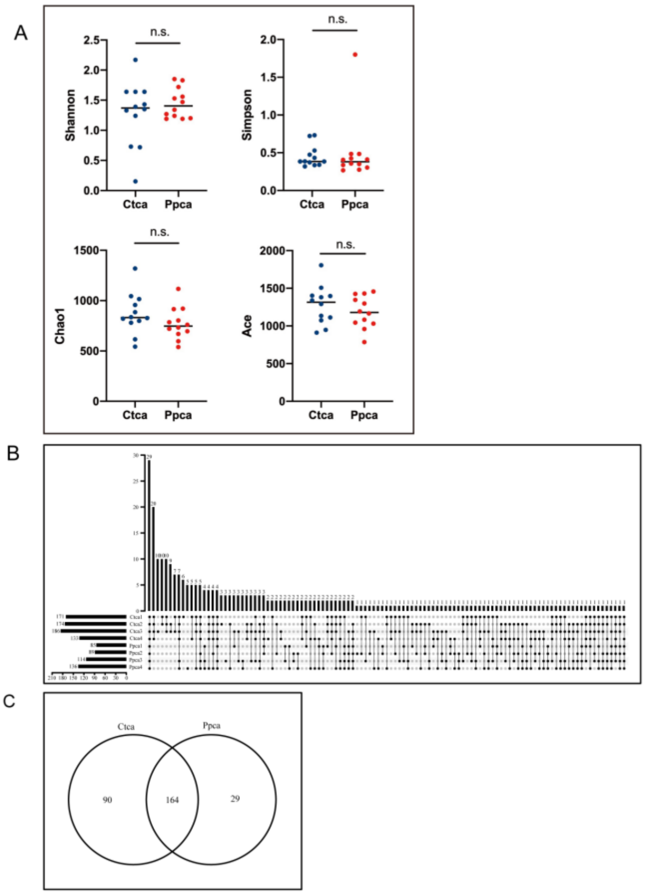

Figure S5 Housefly larvae intestinal bacteria diversity and richness index information and shared and unique genera of the intestinal bacteria in housefly larvae samples. (A) Dynamics of the ACE, Chao1, Shannon and Simpson indices of intestinal bacteria in the two groups of housefly larvae. (B) UpSet plot showing differences in the levels of bacterial genera in the intestinal bacteria of housefly larvae between the two groups within 1-4 days. (C) Venn diagram comparing unique and overlapping bacterial genera in the groups. The numbers represent the numbers of unique genera in each sample and common genera shared by two or more samples.

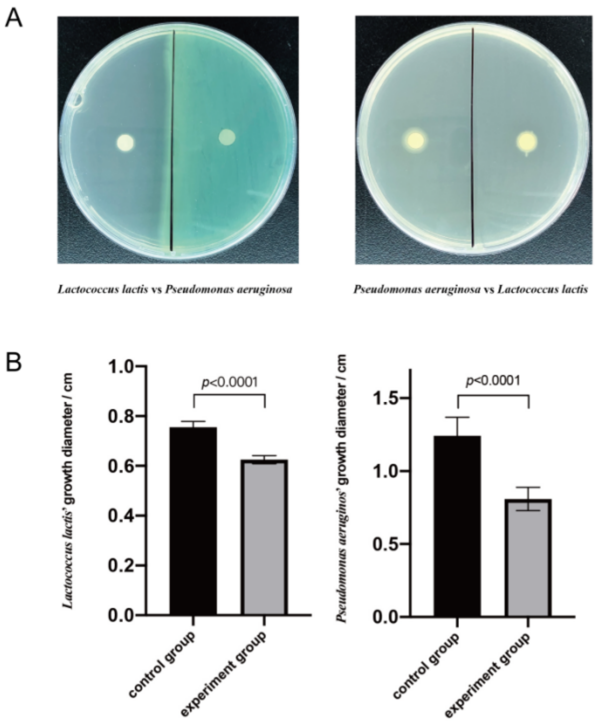

Figure S6 Antagonism experiment of *Pseudomonas aeruginosa* and *Lactococcus lactis* in the housefly larval intestine in a microaerobic environment. (A) Left panel: *P. aeruginosa* was seeded on the right side of the plate, while sterile water was spread on the opposite side as a control. The filters paper was dipped into *Lactococcus lactis*. Right panel: *Lactococcus lactis* was seeded on the right side of the plate, while sterile water was spread on the opposite side as a control. The filter paper was dipped into *P. aeruginosa*. (B) Competitive inhibition between *Pseudomonas aeruginosa* and *Lactococcus lactis* in the housefly larval intestine. Data are shown as the mean ± SEM. The t-test was used for the statistical analysis.
